# Changes to healthcare utilisation and symptoms for common mental health problems over the first 21 months of the COVID-19 pandemic: parallel analyses of electronic health records and survey data in England

**DOI:** 10.1016/j.lanepe.2023.100697

**Published:** 2023-07-21

**Authors:** Vicky P. Taxiarchi, Morwenna Senior, Darren M. Ashcroft, Matthew J. Carr, Holly Hope, Matthew Hotopf, Evangelos Kontopantelis, Sally McManus, Praveetha Patalay, Sarah Steeg, Roger T. Webb, Kathryn M. Abel, Matthias Pierce

**Affiliations:** a Faculty of Biology, Medicine and Health, Division of Psychology and Mental Health, Centre for Women’s Mental Health, University of Manchester, Manchester, UK; b Faculty of Biology, Medicine and Health, Division of Pharmacy and Optometry, The University of Manchester, Manchester, UK; c National Institute for Health and Care Research (NIHR) Greater Manchester Patient Safety Research Collaboration (GM PSRC), University of Manchester, UK; d Department of Psychological Medicine, Institute of Psychiatry Psychology and Neuroscience, King’s College London, London, UK; e Faculty of Biology, Medicine and Health, Division of Informatics, Imaging and Data Sciences, The University of Manchester, Manchester, UK; f Violence and Society Centre, City, University of London, London EC1V 0HB, UK; g Centre for Longitudinal Studies and MRC Unit for Lifelong Health and Ageing, University College London, London, UK; h Division of Psychology & Mental Health, Centre for Mental Health and Safety, The University of Manchester, Manchester, UK; i NIHR School for Primary Care Research, UK

**Keywords:** COVID-19 pandemic, Common mental health problems, Psychological distress, Primary care, Depression, Anxiety, Adults

## Abstract

**Background:**

Few studies have investigated the effect of the COVID-19 pandemic on mental health beyond 2020. This study quantifies changes to healthcare utilisation and symptoms for common mental health problems over the pandemic’s first 21 months.

**Methods:**

Parallel cohort studies using primary care database and survey data for adults (≥16 years) in England from January 2015 to December 2021: 16,551,842 from the Clinical Practice Research Datalink (CPRD) and 40,699 from the UK Household Longitudinal Survey (UKHLS). Interrupted time-series models estimated changes in monthly prevalence of presentations and prescribed medications for anxiety and depression (CPRD); and self-reported psychological distress (UKHLS). The pandemic period was divided into five phases: 1st Wave (April–May 2020); post-1st Wave (June–September 2020); 2nd Wave (October 2020–February 2021); post 2nd Wave (March–May 2021); 3rd Wave (June–December 2021).

**Findings:**

Primary care presentations for depression or anxiety dropped during the first wave (4.6 fewer monthly appointments per 1000 patients, 4.4–4.8) and remained lower than expected throughout follow-up. Self-reported psychological distress exceeded expected levels during the first (Prevalence Ratio = 1.378, 95% CI 1.289–1.459) and second waves (PR = 1.285, 1.189–1.377), returning towards expected levels during the third wave (PR = 1.038, 0.929–1.154). Increases in psychological distress and declines in presentations were greater for women. The decrease in primary care presentations for depression and anxiety exceeded that for physical health conditions (rheumatoid arthritis, diabetes, urinary tract infections). Anxiety and depression prescriptions returned to pre-pandemic levels during the second wave due to increased repeat prescriptions.

**Interpretation:**

Despite periods of distress during the pandemic, we did not find an enduring effect on common mental health problems. The fall in primary care presentations for anxiety or depression suggests changing healthcare utilisation for mental distress and a potential treatment gap.

**Funding:**

10.13039/501100000272National Institute for Health and Care Research (NIHR).

**Research in context**

**Evidence before this study**

Web of Science was searched for meta-analyses, published since March 2020, examining changes in common mental health problems using electronic health records or survey data and included UK studies. Titles were searched for (COVID-19 OR coronavirus OR SARS-CoV-2) AND (((mental OR psych∗) NEAR/1 (disorder OR illness or distress)) OR depression OR anxiety) AND ((“electronic health records” OR “EHR” OR “registry”) OR (“survey”)). This revealed 638 reviews, of which 6 were considered relevant. These reported small but noticeable increases in symptoms of psychological distress, particularly among women, ethnic minorities, parents, and young people. They also reported substantial declines in mental health treatment during the pandemic. However, studies focused on the initial months of the pandemic, leaving a gap in understanding about predicted longer-term effects on mental health and its treatment. The search was implemented on the 6th April 2023.

**Added value of this study**

This study is the first to investigate how the COVID-19 pandemic influenced common mental health problems longer-term in adults in England using both electronic health records and survey data for the first 21 months. Our findings indicate that while presentations to primary care for depression and anxiety remained lower than expected throughout the pandemic, self-reported psychological distress in surveys remained higher than expected up to the end of the 2nd wave, before returning to expected levels after June 2021. We also reveal that people reporting psychological distress were less likely to access mental health treatment in primary care during the pandemic than before.

**Implications of all the available evidence**

This evidence indicates that, whilst the pandemic did not seem to lead to a lasting change in the prevalence of psychological distress, it has changed how people manage their mental health and access mental health care. For some, this may lead to a treatment gap and future research should explore whether the shift to telephone consultations means some are less likely to seek needed treatment for mental health problems. Understanding these factors can inform strategies to improve the quality of prevention measures and treatment for mental health.

## Introduction

Early in the COVID-19 pandemic there was concern that loss of social contacts, fear of the virus, economic shocks and bereavement would result in a significant worsening in population mental health.^1^ In the early months rapid, well-publicised UK surveys^2^^,^^3^ supported this notion and systematic reviews of international studies reported small but noticeable increases in symptoms of psychological distress following the onset of the pandemic, particularly for women, ethnic minorities, parents and young people.^4^^,^^5^ At the same time, specialist mental health services operated at reduced capacity, both in the UK^6^ and elsewhere.^7^ In the UK, where primary care acts as the first-contact for those seeking help for mental health problems, and where the vast majority of psychotropic medications are prescribed, there were sharp declines in contact with GP’s associated with the pandemic, as people were instructed not to burden services.^8^

Beyond the acute phase of the public health emergency, the question for mental health services is: has there been an enduring effect on people’s mental health and an increase in care-seeking? Or has there been a change in the way people manage their mental health, such that fewer people with psychological distress attend primary care? If this is the case, this may indicate a ‘treatment gap’, whereby some who require treatment are put off from seeking it, thereby worsening population mental health. It may also mean people’s symptoms have resolved without treatment, or they found support elsewhere. Answering these questions is crucial if we are to inform recovery from COVID-19 and understand how to respond optimally in future pandemics.

Current evidence about mental health and its treatment beyond the pandemic’s first six months is limited. Some UK surveys reported that population mental health recovered in the summer of 2020^9^ to near pre-pandemic levels; others reported further deterioration during the winter of 2020–2021, as further infection waves occurred.^10^ By September 2020, attendances at primary care for depression and anxiety had returned to similar levels seen pre-pandemic^8^; however self-harm presentations remained lower than pre-pandemic up until at least May 2021.^11^

We examined the longer-term effects of the pandemic on symptoms presentations of, and medication treatment for, common mental health problems over 21 months. We conducted analyses of a large, primary care electronic health record dataset and a nationally representative survey using interrupted time series analysis comparing the observed prevalence of common mental health problems during the pandemic with that predicted by pre-pandemic trends. Triangulating information across two data sources enabled hypotheses to be tested that would not be possible using single sources. There were three aims. For aim 1, we tested the hypothesis that the pandemic resulted in a sustained increase in adults with common mental health problems, evidenced by either high self-reported psychological distress levels, or treatment-seeking for mental health problems. For aim 2, we explored whether, beyond the first 6 months of the pandemic, trends in psychological distress are mirrored in trends in people accessing services. For aim 3, we considered whether trends in treatment-seeking reflected broader changes in healthcare access that affected both mental and physical health; or whether the pandemic had a distinct impact on presentations for mental health problems. To explore this, we examined changes in the ratio of common mental health presentations with presentations for physical health problems, selecting physical health problems that were unlikely to be affected directly by the pandemic, but that *were* likely to have been affected by changes in healthcare access (rheumatoid arthritis, diabetes or urinary tract infections).

## Methods

### Study design

Interrupted time-series analyses quantified the change in symptoms of, and healthcare utilisation for, common mental health problems associated with the COVID-19 pandemic. Psychological distress symptoms were measured in survey data, and primary care presentations and prescriptions for anxiety or depression in electronic health records. The prevalence of each outcome was calculated monthly, from January 2015 to December 2021. Change associated with the pandemic was quantified by comparing the observed prevalence during the pandemic with the estimated counterfactual prevalence, had the pandemic not happened (excluding March 2020, when England transitioned to the pandemic).

### Survey cohort

**Fig. 1:**
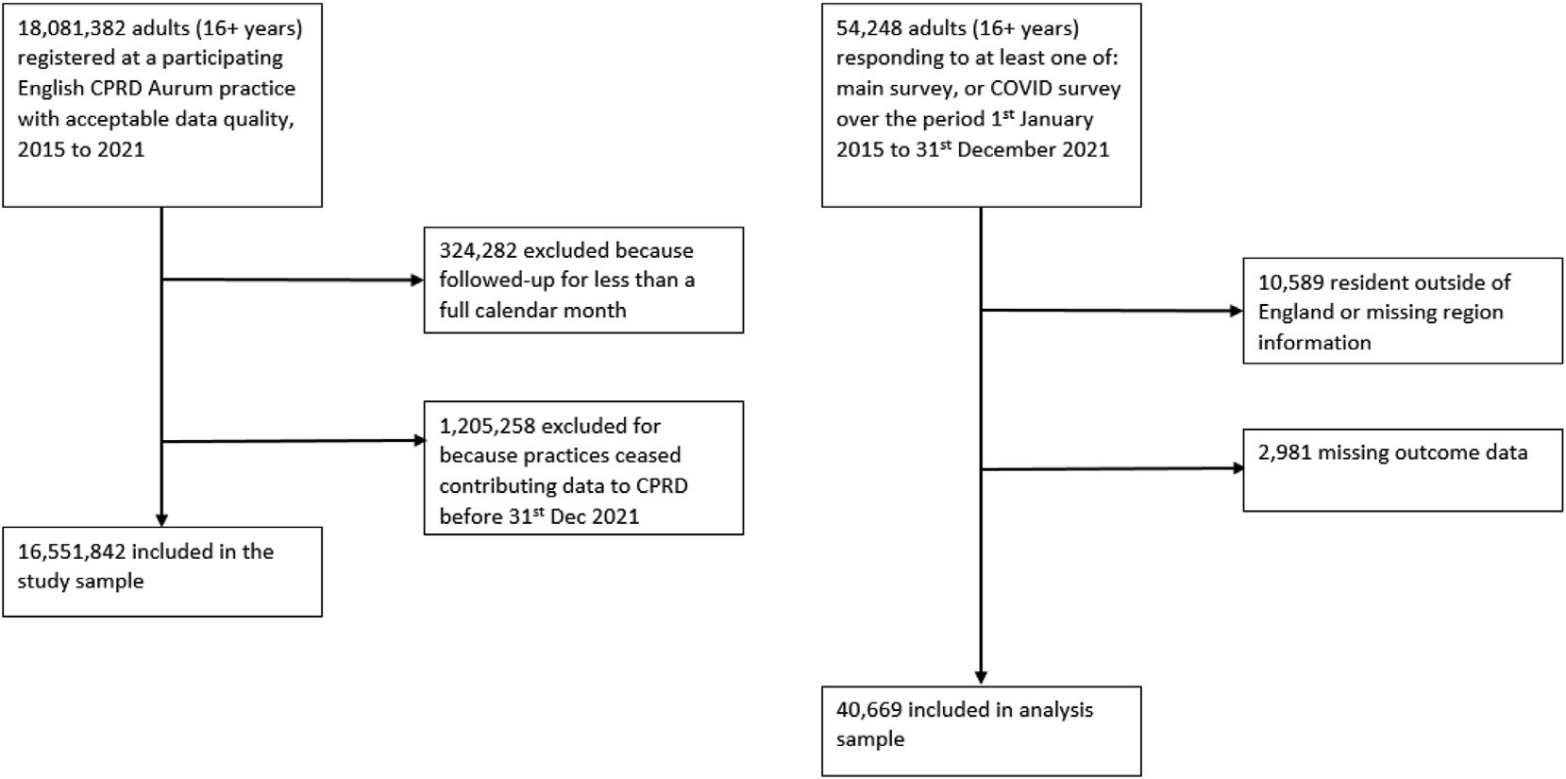
Flow chart representing selection into each cohort.

A cohort of adults (age 16 and over) was identified from the UK Household Longitudinal Survey (UKHLS). The UKHLS is an ongoing, longitudinal survey of approximately 40,000 randomly selected UK households, with yearly data collected through face-to-face interviews. In addition, respondents to the most recent pre-pandemic survey were invited to participate in 9 COVID-19 surveys (conducted online or over the telephone from April 2020 through September 2021; see Appendix pg 2–3 for further details on the UKHLS). Participants were included if they were resident in England and participated in at least one survey over the period January 2015 to December 2021. This resulted in 40,669 in the survey cohort (Fig. 1).

### Primary care cohort

Data on patients, aged 16 and over, registered with an English GP were extracted from the Clinical Practice Research Datalink (CPRD) Aurum dataset.^12^ Aurum holds data on clinical contacts, prescriptions and patient characteristics from GPs that use the EMIS Web® patient system software. Clinical observations and prescriptions are coded using a combination of SNOMED, Read and local EMIS® codes and the Dictionary of Medicines and Devices, respectively. For the May 2022 version (the version used in the analysis), the Aurum database held data on approximately 19.8% of all GP-registered patients in England.^13^ 18,081,382 adults had data of acceptable quality, as per the data custodian’s specifications based on consistently recorded date of birth, practice registration and transfer-out dates,^12^ and were registered at an Aurum-participating practice for at least one calendar month over the study period 1st January 2015 to 31st December 2021.

For the primary care cohort, follow-up started at the latest date of the patient’s: 16th birthday; registration at practice; or 1st January 2015. Their follow-up ended on the earliest date of: patient transferred out from practice; death; or 31st December 2021. Patients were excluded if they were followed-up less than a month (N = 324,382), In addition, a number of practices stopped contributing data to Aurum during the pandemic and exploratory analyses revealed these were a biased subset; therefore to minimise potential selection effects, these patients were excluded (N = 1,205,258). There were 16,551,842 patients in the primary care cohort (Fig. 1).

### Outcomes

For the survey cohort, psychological distress was measured using the 12-item General Health Questionnaire (GHQ-12)^14^; measuring self-reported general mental health, using a 4-point Likert scale. A common mental health problem was defined as scoring in two highest categories in ≥4 questions, in accordance with official NHS statistics.^15^ For the primary care cohort, codes were identified indicating symptoms or diagnosis of depression or anxiety disorder, or prescriptions for anxiolytics, hypnotics or antidepressants. Code lists are published online [https://osf.io/u3d4f/]. In order to exclude non-mental health indications (e.g. antidepressants for neuropathic pain), prescriptions were included when there was a historical diagnosis of anxiety/depression, or a symptom 3 months before/after.

### Covariates

For both data sources, data were extracted on: ethnicity (White, Black, Asian, other); gender (women, men, indeterminate); age (16–24, 25–34, 35–49, 50–64, 65+); regional zone (North, Midlands, South); and quintiles of the Index of Multiple Deprivation (IMD; an area level measure of deprivation; see Appendix pg 4 for further details on definition of covariates). The pandemic period was divided into five phases: 1 = 1st Wave (April–May 2020); P1 = post-1st Wave (June–September 2020); 2 = 2nd Wave (October 2020–February 2021); P2 = post 2nd Wave (March–May 2021); 3 = 3rd Wave (June–December 2021). The definition of these phases was based on infection ‘waves’ reported by ONS,^16^ and the periods of imposed national lockdowns and “three tier system” restrictions.^17^

To investigate whether changes in anxiety or depression presentations differed from physical health problems, consultations for ‘control’ conditions of rheumatoid arthritis (RA), diabetes and urinary tract infections (UTI) were extracted. These were selected based on three criteria: i) it is not plausible that the COVID-19 pandemic resulted in a measureable change in the rate of these diseases; ii) there is demographic overlap between these conditions and anxiety and depression; iii) patient’s with these conditions commonly attend primary care (see Appendix pg 5 for further details).

### Data analysis

Monthly period prevalence was calculated, as the number with outcome divided by the number surveyed (survey cohort, accounting for survey weights) or by the number followed-up for the entirety of that month (primary care cohort). For the primary care cohort, an outcome was defined as at least one occurrence within a calendar month. To examine changes in healthcare utilisation for common mental health problems, relative to control physical health conditions, the ratios of monthly prevalence of anxiety/depression to the monthly prevalence of RA, diabetes, and UTI were calculated as adjusted measures, to account for different ratios according to subgroups (e.g. older people experiencing higher rates of diabetes, lower rates of anxiety) and that the pandemic had a differential effect on healthcare access.^18^ The ratios were calculated within subgroups of ethnicity, age and gender and subsequently combined using a weighted sum, according to the sample size in each subgroup.

Binomial GLM’s with a logit link function were fitted to monthly pre-pandemic data (January 2015 to February 2020) and used to predict the expected prevalence during the pandemic (April 2020 to December 2021). Models included variables for: seasonality; period trends; and autocorrelation (model building algorithm detailed in Appendix pg 6). Effect sizes were the rate difference (observed minus expected) or the rate ratio (observed divided by expected). Models were fitted to subgroups: age, gender, region, ethnicity and IMD quintile; excluding missing subgroups. Missing data were infrequent (<0.2%), except for ethnicity in the primary care cohort (7.7%). Confidence intervals were calculated using the bootstrap technique, from 2.5th and 97.5th percentiles of 1000 random samples. The bootstrap procedure accounted for clustering both at individual-level and, for the survey cohort, within households. As a result of the computational burden of this procedure, samples were constrained to no more than 1 million individuals.

### Sensitivity analyses

Two sensitivity analyses considered whether results were robust to changes in coding practices by repeating the analysis with the outcome defined by: (i) diagnostic codes only; or (ii) diagnostic codes, or symptom codes with a prior diagnosis. A further sensitivity examined whether the analysis was robust to the choice of model, therefore autoregressive integrated moving average (ARIMA) models were fitted.

### Post hoc analyses

Additional analyses, not part of the initial analysis plan, examined trends of new and repeated presentations or prescriptions. To calculate new presentations/prescriptions, the analysis was restricted to those without the outcome within six months prior to the analysis month. For repeated events, the analysis was restricted to those with an outcome event within six months prior.

Data management and analyses were carried out in Stata MP v16, and the graphs were created using R version 4.2.2.

### Role of the funding source

None.

## Results

There were 16,551,842 adults (aged 16 years and older) in the primary care cohort (median follow-up = 4.4 years, IQR 1.7–7.0), and 40,699 in the survey cohort (median follow-up = 4.8 years, IQR 1.6–5.9). There were similar proportions of women in both (51.3% in the primary care cohort and 50.3% in the survey after weighting; Table 1). In the primary care cohort, there were more people from non-white ethnic groups (19.6% versus 11.8%) and from the South of England (59.3% versus 51.9%).

**Table 1:** Characteristics of the two study cohorts.

	Primary care cohort (N = 16,551,842)		Survey cohort^a^ (N = 40,669)		
	N	%	N	% unweighted	% weighted
**Gender**					
Female	8,485,341	51.3	22,016	54.1	50.3
Male	8,065,832	48.7	18,644	45.9	49.7
Indeterminate	669	0.0	–	–	–
Missing	–	–	9	–	–
**Year of birth**					
<1950	2,346,301	14.2	6,888	16.9	19.4
1950–<1964	2,646,404	16.0	8,505	20.9	21.4
1965–<1979	3,573,678	21.6	10,264	25.2	22.6
1980–<1989	3,310,584	20.0	6,072	14.9	14.2
1990–2005	4,674,875	28.2	8,882	21.8	22.4
**Ethnicity**					
Asian	1,338,953	8.8	6,509	16.0	6.4
Black	760,487	5.0	2,633	6.5	2.6
White	12,284,412	80.4	29,834	73.5	88.2
Other	888,012	5.8	1,636	4.0	2.6
Missing	1,279,978	–	57	–	–
**IMD quintile**					
1 (most deprived)	3,109,838	18.8	10,125	24.9	18.9
2	3,226,687	19.5	8,267	20.3	18.9
3	3,277,803	19.8	7,679	18.9	20.6
4	3,711,447	22.4	7,481	18.4	21.2
5 (least deprived)	3,226,048	19.5	7,117	17.5	20.5
Missing	19	–	–	–	–
**Regional zone**					
North	3,837,776	23.2	11,394	28.0	28.8
Midlands	2,906,328	17.6	7,943	19.5	19.3
South	9,807,738	59.3	21,332	52.5	51.9

Missing data excluded from percentages.

IMD = Index of Multiple Deprivation.

a Characteristics taken from the earliest wave.

### Association between COVID-19 pandemic and psychological distress and healthcare utilisation for anxiety or depression

**Fig. 2:**
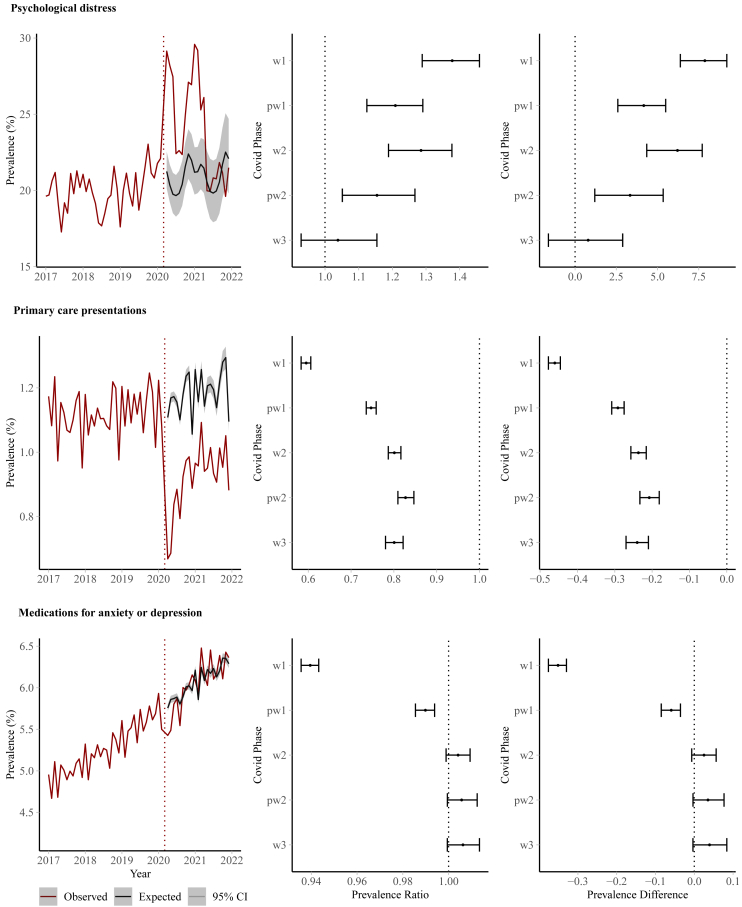
Trends in common mental health problem symptoms and utilisation over the pandemic for outcomes: (first row) psychological distress, (second row) primary care presentations for anxiety or depression, (third row) medications for anxiety or depression, showing (first column) time-series of observed and expected trends, (second column) risk ratios and (third column) risk differences, according to phases of the pandemic: 1 (April–May 2020); P1 (June–September 2020); 2 (2nd wave October 2020–February 2021); P2 (March–May 2021); 3 (June–December 2021).

There was evidence of a sustained increase in psychological distress between April 2020 and May 2021 compared to pre-pandemic trends (Fig. 2). This was particularly evident during the first wave (Prevalence Ratio, PR = 1.378, 95% Confidence interval 1.289–1.459; Prevalence difference, PD = 7.87%, 6.38–9.20%) and second wave (PR = 1.285, 1.189–1.377; PD = 6.21%, 4.35–7.71%). By the third wave (June–December 2021) there was little evidence of increase (PR = 1.038, 0.929–1.154; Appendix pg 7).

Compared to pre-pandemic trends, presentations to primary care with anxiety or depression dropped substantially during the pandemic (Fig. 2); particularly during the first wave of the pandemic (April–May 2020) when monthly prevalence was 40% (39–42%) lower than expected (4.6 fewer appointments per 1000 patients, per month, 95% CI 4.4–4.8). The rate remained lower than expected up to December 2021. For example, there were 20% fewer appointments during the third wave (June–December 2021) and declines were observed irrespective of whether the appointment was for an anxiety or depression (Appendix pg 7). Medications prescribed for treating anxiety or depression were lower than expected during the first wave of the pandemic (PR = 0.940, 0.936–0.943), but returned to pre-pandemic levels during the second wave (2nd wave PR = 1.004, 0.999–1.009). When results were disaggregated according to medication type, there was evidence of a marginal increase in antidepressant prescriptions in the second wave and beyond (Appendix pg 7).

### Subgroup analyses

**Fig. 3:**
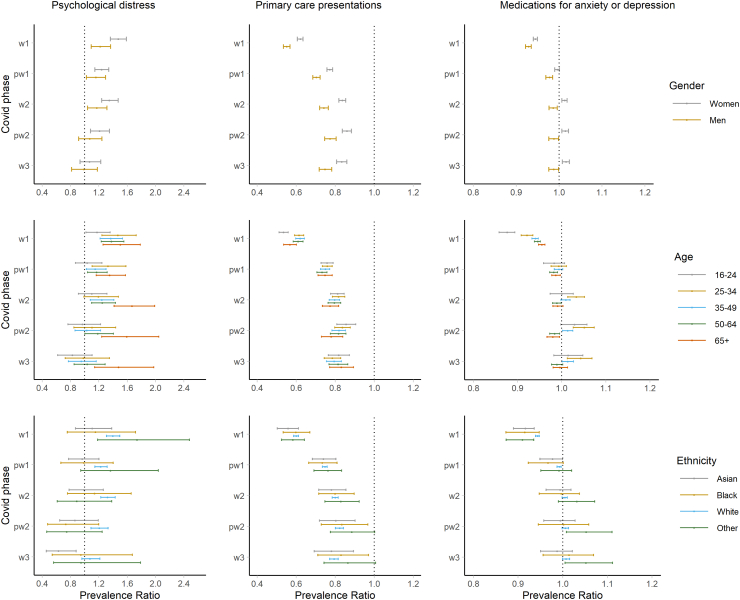
Prevalence Ratio (PR) between observed and expected values for subgroups, by each outcome (psychological distress, primary care presentations for anxiety or depression, medications for anxiety or depression medications); by pandemic phase (1 (April–May 2020); P1 (June–September 2020); 2 (October 2020–February 2021); P2 (March–May 2021); 3 (June–December 2021)).

Based on pre-pandemic trends, women had a greater increase in self-reported psychological distress during most phases of the pandemic, particularly during the 1st wave, when an additional 11.2% of women (9.0–13.0%) experienced psychological distress, compared to 3.9% of men (1.8–6.1%). Women had a smaller relative reduction in primary care presentations for anxiety or depression during the pandemic than men (e.g. during the first wave women PR = 0.621, 0.606–0.635; men PR = 0.551, 0.535–0.568, Fig. 3, Appendix pg 10–12) but a larger absolute reduction (e.g. first wave, women RD = −0.54%, −0.57 to −0.52% and men RD = −0.38%, −0.40 to 0.36%). During the pandemic’s second phase and beyond, there was a small increase in the proportion of women being prescribed medications for common mental health problems compared with the expected rate, whereas there was a small decrease in the proportion of men. For example, during 2nd wave (October 2020–February 2021) 0.10% (0.05–0.15%) more women were prescribed medications for common mental health problems, whereas 0.06% (0.02–0.09%) fewer men were.

The decline in presentations for common mental health problems during the pandemic’s 1st wave was more pronounced for 16–24 years old than others (Fig. 3; Appendix pg 13–17). Those aged 25–34 saw increases in prescriptions during the 2nd wave or later. Psychological distress levels recovered for adults of 25–64 years old during the 3rd wave, whereas they increased for older adults (65+) and remained higher up until December 2021.

Although analyses stratified by ethnicity were underpowered for some groups, higher than expected distress levels were observed for Asian people during the 1st wave, and in White people in all waves (Fig. 3; Appendix pg 18–21). There was no notable difference in the patterns of distress or healthcare utilisation according to IMD (Appendix pg 22–27) and regional zone (Appendix pg 27–31), although there was evidence of higher than expected distress levels within the 4th quintile (where the 5th quintile represents the most deprived areas) and in the Midlands.

### Comparison with physical health problems

**Fig. 4:**
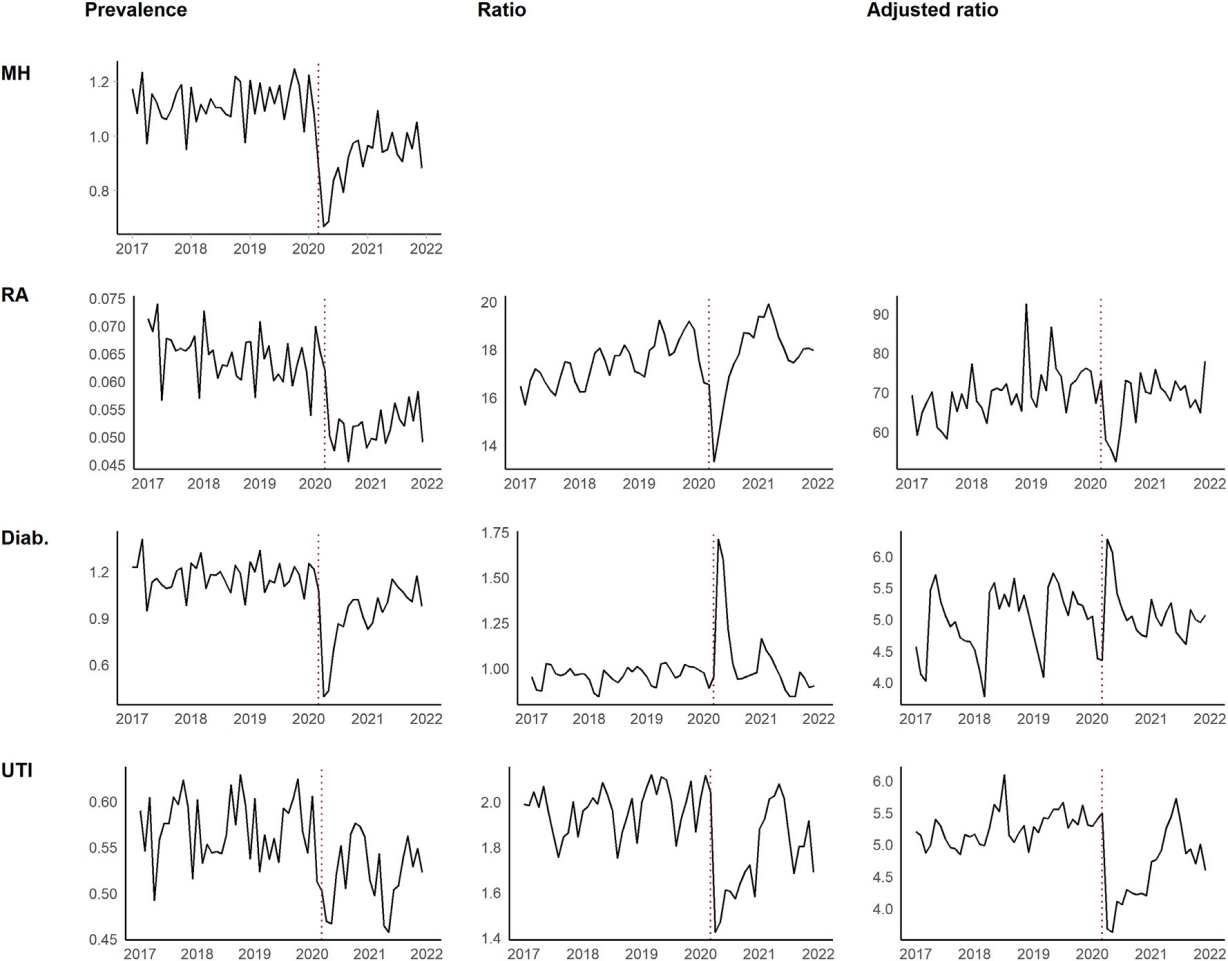
Presentations to primary care for (first row) anxiety or depression; (second row) rheumatoid arthritis; (third row) diabetes; and (forth row) urinary tract infections, showing: (first column) monthly prevalence of condition, (second column) ratio between condition and presentations for common mental health problems (third column) ratio adjusted for patient demographics.

During the pandemic, there were sharp declines in primary care presentations for diabetes, RA and UTI’s (Fig. 4). The ratio between presentations for common mental disorders and those for RA and UTI’s fell during the first few months of the pandemic, indicating a greater decline for common mental health problems. During the first wave, the drop in presentations was greater for diabetes. This was explained partly by differences in patient demographics in this group, as demonstrated by the smaller increase in the adjusted ratio.

### Sensitivity and post-hoc analyses

Very similar results were obtained when primary care presentations were restricted to diagnoses only, or symptoms where there was a history of a diagnosis (Appendix pg 32–33). Results were also similar when ARIMA models were used (Appendix pg 34). In post-hoc analyses, a sustained decrease was observed in incident prescriptions (those without prescriptions in the previous 6 months) and a small increase in repeat prescriptions (those with prescriptions in the previous 6 months Appendix pg 35–36).

## Discussion

Amongst adults in England, presentations to primary care for depression and anxiety remained lower than expected throughout the first 21 months of the COVID-19 pandemic, whilst psychological distress reported in surveys remained higher than expected up to the end of the 2nd wave. Importantly, psychological distress reported in surveys returned to expected levels after June 2021, coinciding with the point where most adults were fully vaccinated and social restrictions were stopped.^19^ This indicates that our initial hypothesis (aim 1), that the pandemic had an enduring effect on common mental disorders, is not borne out in the data and that population mental health was resilient to the effects of the pandemic.

Our findings suggest that the trends in mental health problems during the pandemic was not mirrored in the trends in presentations to primary care (aim 2). It follows that fewer people with psychological distress presenting to primary care. This phenomenon is illustrated by considering the ratio between the monthly prevalence of primary care presentations to the monthly prevalence of reported psychological distress. During the first wave this ratio was expected to be 0.055, as predicted by pre-pandemic trends; whereas the observed value was less than half that at 0.024. If we assume that all those who presented to primary care with symptoms of anxiety or depression had measureable psychological distress, we can infer that, during the first wave, out of those with psychological distress, the pandemic was associated with 3.1% fewer, per month, not presenting to primary care (3.5% of women and 2.6% of men). This difference reduced to 1.4% of women and 1.3% of men during the 3rd wave.

Overall, there were reductions in the proportion of adults prescribed medications for symptoms of anxiety or depression. When considered alongside sharp declines in the availability of face-to-face talking therapies during the pandemic,^20^ there appears to be no evidence of a shift from psychological to pharmacological treatment, as has been suggested.^21^ However, we reveal that, whilst there were fewer new prescriptions than expected, there were increases in repeat prescriptions; possibly because fewer people sought advice from their GPs about how to come off their medications. Alternatively, it could signify that some felt less able to stop their medications, given the challenges of the pandemic and perceived or real inaccessibility of GPs.

Consistent with prior evidence,^3^^,^^9^ we report that women’s mental health declined more steeply than men’s during the pandemic. This is likely because women were disproportionately disadvantaged by pandemic measures: school closures meant women took on more child care and domestic responsibilities^22^; and lockdowns resulted in greater rates of domestic violence^23^; women were also more likely to lose out economically, widening existing economic gender inequalities.^24^ Women also had a larger absolute decrease in presentations to primary care for mental health problems. This may mean women are particularly vulnerable to a treatment gap, such that their mental health needs were not being met during the pandemic. Alternatively, more women may previously have presented with distress that did not require medical intervention and resolved spontaneously.

We also report that the decline primary care presentations for depression and anxiety was greater than the decline in presentations for physical conditions unlikely to be affected by the pandemic (aim 3). We made these comparisons whilst controlling for age, gender and ethnicity, therefore the results suggests there is something specific about patients with mental health presentations, independent of their demographics, that meant they were less likely to attend primary care during the pandemic. One reason behind this could be the shift to primary care telephone consultations–the ratio of telephone to face-to-face appointments changing from 1:5.8 in February 2020 to 1:1.8 by February 2022.^25^ It could also be that less severe, more common mental health symptoms may find alternative sources of non-medical support more readily than some physical health problems that require medical help.

There are several limitations. First, for parameter estimates to identify the effect of the pandemic, the fitted model must describe the pre-pandemic trend correctly. We ascertained the robustness of the findings to this assumption by fitting ARIMA models, which have different model specifications. Second, it must be assumed that the trend that occurred prior to March 2020 is generalisable to the trend after April 2020, had the pandemic not happened. To mitigate this, we used a test dataset in our model building, to find a model that had good out-of-sample prediction. Third, each data source imperfectly measures mental health problems. Primary care data can ascertain treatment access but cannot separate treatment-seeking from the true clinical need in the community. We attempted to untangle these by comparing our results with ‘control’ conditions, unlikely to be affected directly by the pandemic. Whilst the GHQ-12 is a useful screening tool for depression or anxiety disorders,^26^ it is imprecise and thresholds of self-reported mental health symptom scores tend to overestimate mental disorder prevalence.^27^ In addition, whilst we are able to indirectly examine how primary care visits for those with psychological distress changed due to the pandemic, linked datasets with both outcomes would be preferable. A further limitation is that generalisability of results is affected by which primary care practices submit data to the CPRD dataset and who responds to the UKHLS survey. Relatedly, those who contribute ongoing data may form a selective group and therefore the results may be subject to selection effects due to differential attrition. Also, whilst primary care is the main source of mental health care for most mental health problems in the UK, several other forms of mental health support were not considered; for example talking therapies, or specialist mental health care. Finally, there are multiple hypotheses tested in this study and further confirmatory analyses (preferably in other countries) may be needed to rule out whether some of the findings arose by chance.

### Implications and future research

This study has two important implications for policymakers. First, whilst mental distress increased in the short term during the first two waves of the pandemic (as per the survey dataset), adults in England largely appeared to be psychologically resilient to the pandemic and mental distress returned to pre-pandemic levels. In addition, there was no subsequent ‘surge’ in the numbers of people either seeking assessment and treatment for common mental health symptoms or receiving new prescriptions. It is also of note that other studies have not shown an increase in other markers of mental health, for example: severe mental health symptoms left untreated^28^; or self-harm^11^ or suicide.^29^ In our view, this dispels previous concerns that the pandemic caused a ‘tsunami’ of mental illness.^30^ The second key implication is that the pandemic appears to have changed the ways in which people manage their mental health and subsequently access mental health care. For some, this may represent a widening treatment gap, whereby people who need treatment are no longer presenting to services. However, more treatment does not necessarily translate into a smaller treatment gap, which might be better narrowed by improving prevention measures and the quality of treatment.^31^ Furthermore, for others, perhaps especially women, it may mean a reduction in use of medical services for milder, more transient problems.

Future research should determine whether the changing way in which the pandemic has led to delivery of care has led to greater unmet clinical need, because people are inappropriately put off using alternative systems, e.g. telephone consultations for mental health problems. It should also clarify whether a reduction in face-to-face healthcare means more people will be on fewer, or more, long-term psychotropic medications. This is important given patient preferences for less pharmacological and more psychological approaches to mental health treatment.^32^ In our view, such future research would greatly benefit from broad stakeholder involvement to consider the risks and benefits of a changing landscape for mental healthcare delivery.

## Contributors

MP conceived of the study and wrote the initial analysis plan, and all authors (except MH) contributed. MC extracted the data. MS and KMA finalised clinical indications. MP and VPT had access to and verified the data, and conducted the analyses. All authors contributed to data interpretation. MP and VPT wrote the initial draft and created tables and figures. All authors contributed to the final manuscript and approved its submission for publication.

## Data sharing statement

Clinical code lists and data management code are published on osf.org. Electronic health records are, by definition, considered “sensitive” data in the UK by the Data Protection Act and cannot be shared via public deposition because of information governance restriction in place to protect patient confidentiality. Access to data is possible only once approval has been obtained through the individual constituent entities controlling access to the data. The primary care data can be requested via application to the Clinical Practice Research Datalink (www.cprd.com/researcher), secondary care data can be requested via application to the hospital episode statistics from the UK Health and Social Care Information Centre (www.hscic.gov.uk/hesdata).

## Ethics approval

The study was approved by the Independent Scientific Advisory Committee (ISAC) for MHRA Database Research (protocol number: 20_094). Generic ethical approval for the CPRD has been granted by a Health Research Authority (HRA) Research Ethics Committee (East Midlands—Derby, REC reference number 05/MRE04/87).

## Declaration of interests

MH is Principal Investigator of the RADAR-CNS consortium, a precompetitive public-private partnership on mobile health, funded by Innovative Medicines Initiative. The partnership includes research funding contributions from Janssen, Lundbeck, UCB, Biogen and MSD.
