# Supplementary material for: Changes to healthcare utilisation and symptoms for common mental health problems over the first 21 months of the COVID-19 pandemic: parallel analyses of electronic health records and survey data in England
Source: Lancet Reg Health Eur. 2023 Jul 21;32:100697. doi: 10.1016/j.lanepe.2023.100697 (PMC10477036; doi:10.1016/j.lanepe.2023.100697)
Supplement: Appendix [file mmc1.docx]

**Supplementary Material**

**Table of Contents**

[Further details on the UK Household Longitudinal Study 2](#_Toc137216198)

[Table S1: The Understanding Society mainstage waves 5-12 and the 9 COVID-19 waves 2](#_Toc137216199)

[Supplementary Table S2: Further information on definition of covariates in each dataset 4](#_Toc137216200)

[Selection of control conditions 5](#_Toc137216201)

[Model building algorithm 7](#_Toc137216202)

[Supplementary Table S3: results for whole cohort, by primary outcome measures 8](#_Toc137216203)

[Supplementary table S4: results by secondary outcome measures 9](#_Toc137216204)

[Supplementary Figure S1: plots by secondary outcome measures 10](#_Toc137216205)

[Supplementary table S5: results by gender 11](#_Toc137216206)

[Supplementary Figure S2: time-series plots by gender 13](#_Toc137216207)

[Supplementary table S6: results by age-group 14](#_Toc137216208)

[Supplementary Figure S3: time-series plots by age-group 18](#_Toc137216209)

[Supplementary table S7: results by ethnicity 19](#_Toc137216210)

[Supplementary Figure S4: time-series plots by ethnicity 22](#_Toc137216211)

[Supplementary table S8: results by IMD quintile 23](#_Toc137216212)

[Supplementary Figure S5: time-series plots by IMD quintile 27](#_Toc137216213)

[Supplementary Figure S7: Time-series plots by region 32](#_Toc137216214)

[Supplementary Table S10: Sensitivity analysis 1 changing definition of outcome in primary care cohort 33](#_Toc137216215)

[Supplementary Figure S8: plots after changing definition of outcome in primary care cohort 34](#_Toc137216216)

[Supplementary Figure S9: Sensitivity 2 Results from time-series analyses using the ARIMA model 35](#_Toc137216217)

[Supplementary Table S11: post-hoc analysis, results for new and repeated prescriptions and presentations 36](#_Toc137216218)

[Supplementary Figure S10: time-series plots of new and repeated presentations and prescriptions 37](#_Toc137216219)

[References 38](#_Toc137216220)

# Further details on the UK Household Longitudinal Study

The UK Household Longitudinal Study (UKHLS; also called ‘Understanding Society’) began in 2009 as a continuation of the British Household Panel Survey (BHPS) which started in 1991. At the first wave of data collection (Jan 2009 to Mar 2011), the UKHLS included: (i) a clustered and stratified, probability sample of about 24,000 households from the general population sample (GPS) in Great Britain, (ii) a random sample of about 2,000 households from the GPS in Northern Ireland, and (iii) the Ethnic Minority Boost Sample (EMBS) with approximately 4,000 households where at least one member was from an ethnic minority group. At the second wave (Jan 2010 to Mar 2012), about 8,000 households that were earlier participating to BHPS had accepted to join the UKHLS. Finally, in the 6th wave of UKHLS (Jan 2014 to May 2016), an additional sample of about 2,900 households were invited to participate where at least one member was an born out of the UK or was from an ethnic minority group –the Immigrant and Ethnic Minority Boost Sample (IEMBS).

Individuals are no longer followed-up if moved out of the UK, and new participants are added in the study if they become a member of an already participating household. The EMBS and the IEMBS were sampled from areas with high concentration of people from ethnic minority groups. Together with the GPS and the BHPS samples they produced an oversample of ethnic minorities, which is not reflective of the structure of the UK population. To account for design elements and attrition bias, cross-sectional survey weights are calculated by the data custodians. These are based on design weights that account for the stratified sampling scheme and non-response weights, based on the characteristics of those households that did not respond. These weights are then calibrated to be representative of the UK population.

More frequent waves were introduced at the emergence of the COVID-19 pandemic. 9 waves were implemented from April 2020 up to September 2021. Individuals invited to participate in the COVID-19 waves were: from households with known addresses or postal code; who had responded to at least one for the previous two waves; excluding those who were mentally or physically unable to respond.

Further information for each wave regarding the sample size of each wave included in the present study, the data collection period and the mode of data collection is provided below in Table S1.

# Table S1: The Understanding Society mainstage waves 5-12 and the 9 COVID-19 waves

| **Waves** | **Survey period** | **Mode of collection** | **Sample invited** | **Sample responded** | **Response rates (%)** |
| --- | --- | --- | --- | --- | --- |
| Mainstage 5 | Jan 2013 - Jun 2015 | f2f/ phone | 62,307 | 41,041 | 65.9 |
| Mainstage 6 | Jan 2014 - May 2016 | f2f/ phone | 65,031 | 42,021 | 64.6 |
| Mainstage 7 | Jan 2015 - May 2017 | f2f/ web/ phone | 59,101 | 39,092 | 66.1 |
| Mainstage 8 | Jan 2016 - May 2018 | f2f/ web/ phone | 55,053 | 37,565 | 68.2 |
| Mainstage 9 | Jan 2017 - May 2019 | f2f/ web/ phone | 51,484 | 34,959 | 67.9 |
| Mainstage 10 | Jan 2018 - May 2020 | f2f/ web/ phone | 46,761 | 33,514 | 71.7 |
| Mainstage 11 | Jan 2019 - May 2021 | f2f/ web/ phone | 43,831 | 31,542 | 72.0 |
| Mainstage 12 | Jan 2020 - May 2022 | f2f/ web/ phone | 42,029 | 29,070 | 69.2 |
| COVID-19 1 | Apr 2020 | web only | 42,207 | 17,761 | 42.1 |
| COVID-19 2 | May 2020 | web/ phone | 42,182 | 14,811 | 35.1 |
| COVID-19 3 | Jun 2020 | web only | 42,171 | 14,123 | 33.5 |
| COVID-19 4 | Jul 2020 | web only | 42,102 | 13,754 | 32.7 |
| COVID-19 5 | Sep 2020 | web only | 42,080 | 12,876 | 30.6 |
| COVID-19 6 | Nov 2020 | web/ phone | 42,030 | 12,035 | 28.6 |
| COVID-19 7 | Jan 2021 | web only | 42,004 | 11,968 | 28.5 |
| COVID-19 8 | Mar 2021 | web only | 41,967 | 12,680 | 30.2 |
| COVID-19 9 | Sep 2021 | web only | 41,831 | 12,818 | 30.6 |

# Supplementary Table S2: Further information on definition of covariates in each dataset

| **Covariate** | **Definition** |  |
| --- | --- | --- |
|  | **Primary care data** | **Survey data** |
| Gender | Self-defined at registration to primary care. Coded as male, female, or indeterminate. ‘Indeterminate’ means they were unable to be classified as male or female.^9^ | Self-reported in survey. Coded as female, male or missing. |
| Year of birth | Year of birth at registration. Used to determine participant’s age. The CPRD does not disclose month and day of birth, therefore it was assumed that date of birth fell on the 1^st^ July on their year of birth. | Year of birth. |
| Ethnicity | Self-reported at each visit. Ethnicity assigned following previously published algorithm.^10^ Invalid ethnicity code in the CPRD dataset were replaced ethnicity code in linked HES dataset, if valid. | Self-reported in survey. |
| IMD* quintile | Derived from the HES dataset, based on patient’s postcode, in 2019. Available as a linked dataset to CPRD.  Missing values were replaced with the imd as defined in the CPRD data, based on the practice’s postcode, in 2015. | Derived from each household’s postcode. |
| Region | Geographical region of patient’s residence | Geographical region of household’s residence |

*IMD = index of multiple deprivation: A low-level area measure of deprivation (approximately 1500 people), quintiles of the Index of Multiple Deprivation (IMD), was mapped to each general practice for the primary care cohort or household for the survey cohort. ^11^

# Selection of control conditions

For the selection of the control conditions, we considered common diseases with the aim of identifying multiple conditions across different systems (e.g. endocrine, musculoskeletal, genitourinary), which constituted a mixture of both acute and chronic health problems, reflecting the likely case mix of presentations for common mental disorder. We considered the following questions in relation to these conditions:

Is it considered plausible that the pandemic caused a change in the rate of the disease in the population?

Is there overlap in the demographics that are affected by common mental disorders and these diseases? Most importantly, are the diseases incident amongst younger people of both genders?

Would patients commonly attend primary care for this condition?

| **Disease** | **Include?** | **Rationale** |
| --- | --- | --- |
| Asthma | No | Potential for miss-attribution of presentations with respiratory symptoms due to covid. Early in the pandemic there was concern that covid-19 might cause acute exacerbation of asthma.^1^ Incidence of asthma exacerbations potentially fell during the pandemic due to lower pollution levels and lower incidence of non-covid respiratory tract infections.^2^ |
| COPD | No | COPD associated with increased risk of severe covid-19 infection, potentially leading to increase in GP consultations.^2,3^  Low prevalence in young adults. |
| Respiratory tract infections | No | Incidence of RTI’s other than Covid-19 fell due to lower social mixing.^4^  Presentations with respiratory symptoms due to covid may be recorded as generic respiratory tract infections. |
| Hypertension | No | Low prevalence/incidence in young adults.^5^  Often asymptomatic, so cases seen in primary care are likely to have been identified through routine monitoring or as an incidental finding unlike common mental disorder presentations which are expected to be driven by patients seeking help for symptoms. |
| Ischaemic Heart Disease | No | Low prevalence/incidence in young adults |
| Diabetes | Yes | Although COVID-19 infection is associated with subsequent diabetes,^6^ we considered that evidence of a direct causal link was not established, and if there was a link, the effects on the population level for the time-period considered would be relatively small.  GP contacts likely to reflect a mix of acute symptom-driven presentations and ongoing monitoring, similar to common mental disorders. |
| Rheumatoid arthritis | Yes | There is little evidence that COVID infection causes increased risk of RA.^7^ Although it could be hypothesised that there are small effects of lockdowns on flare-ups of RA, due to lower rates of exercise during lockdown. We anticipated that the effects of this were small.  Most common inflammatory arthritis. Compared to other arthropathies (eg. Osteoarthritis), relatively high prevalence in younger adults and incidence considered less likely to be impacted by changing activity levels during lockdown. |
| Back pain | No | Common reason for primary care consultation in adults of both genders, but incidence likely to be impacted by changes in activity levels during lockdowns. |
| Urinary Tract Infections | Yes | High incidence in adults (particularly women), good overlap with demographics of those with common mental disorders.^8^  It was speculated that incidence of this may have fallen due to changes in sexual behaviour, however we considered these effects are likely to be small. |

# Model building algorithm

Generalised linear regression models (GLMs) were fitted to monthly pre-pandemic data, assuming a binomial distribution and a logit link function, from January 2015 to February 2020, and then used to predict each outcome during the pandemic (April 2020 to December 2021).

All models included a linear term for month of follow up. Model building proceeded as follows:

1. two methods were compared to capture seasonality: an indicator variable for month; or three pairs of Fourier terms (three sine and three cosine functions^12^)
2. To capture non-linear trends, squared and cubed terms for month of follow-up
3. partial autocorrelation plots of the deviance residuals were examined, and when there was evidence of autocorrelation, lagged dependent variables with lags of one to three periods were added, or the logarithmic of these.

Model selection was based on the AIC and the ability of the model to predict 21 months of data. To assess the latter, each model was fitted on training data from January 2015 to May 2018 and used to predict outcomes in test data, from June 2018 to February 2020. The observed and expected counts were compared graphically and using the Root Mean Square Error.

Final models were used to predict the expected outcome in each pandemic month, had the pandemic not occurred. The expected rate during each pandemic phase was obtained by dividing the total number of expected events by the total number of patient months. This was contrasted with the observed rate of events, to estimate either the rate difference (observed minus expected) or the rate ratio (observed divided by expected).

# Supplementary Table S3: results for whole cohort, by primary outcome measures

| **Cohort** | **Period** | **Outcome** | **Observed*** | **Expected*** | **Difference**  **[95% CI]** | **Ratio**  **[95% CI]** | **P value** |
| --- | --- | --- | --- | --- | --- | --- | --- |
| Survey | 1^st^ Wave | Psychological distress | 28.70 | 20.83  [19.82, 22.07] | 7.87  [6.38, 9.20] | 1.378  [1.289, 1.459] | <0.001 |
|  | Post 1^st^ Wave |  | 24.09 | 19.92  [18.81, 21.17] | 4.17  [2.59, 5.49] | 1.209  [1.124, 1.291] | <0.001 |
|  | 2^nd^ Wave |  | 27.99 | 21.78  [20.44, 23.20] | 6.21  [4.35, 7.71] | 1.285  [1.189, 1.377] | <0.001 |
|  | Post 2^nd^ Wave |  | 24.94 | 21.60  [20.05, 23.27] | 3.34  [1.19, 5.34] | 1.154  [1.052, 1.267] | 0.004 |
|  | 3^rd^ Wave |  | 21.47 | 20.67  [18.72, 22.84] | 0.79  [-1.62, 2.89] | 1.038  [0.929, 1.154] | 0.249 |
| Primary care | 1^st^ Wave | Presentations for anxiety or depression | 0.68 | 1.14  [1.12, 1.15] | -0.46  [-0.48, -0.45] | 0.595  [0.583, 0.606] | <0.001 |
|  | Post 1^st^ Wave |  | 0.86 | 1.15  [1.14, 1.17] | -0.29  [-0.31, -0.28] | 0.747  [0.735, 0.759] | <0.001 |
|  | 2^nd^ Wave |  | 0.95 | 1.19  [1.17, 1.21] | -0.24  [-0.26, -0.22] | 0.801  [0.787, 0.816] | <0.001 |
|  | Post 2^nd^ Wave |  | 0.99 | 1.20  [1.18, 1.23] | -0.21  [-0.23, -0.18] | 0.827  [0.809, 0.847] | <0.001 |
|  | 3^rd^ Wave |  | 0.96 | 1.20  [1.18, 1.23] | -0.24  [-0.27, -0.21] | 0.801  [0.781, 0.822] | <0.001 |
|  | 1^st^ Wave | Medications for anxiety or depression | 5.46 | 5.81  [5.77, 5.85] | -0.35  [-0.38, -0.33] | 0.940  [0.936, 0.943] | <0.001 |
|  | Post 1^st^ Wave |  | 5.80 | 5.86  [5.83, 5.91] | -0.06  [-0.09, -0.04] | 0.990  [0.986, 0.994] | <0.001 |
|  | 2^nd^ Wave |  | 6.04 | 6.01  [5.97, 6.06] | 0.02  [-0.01, 0.06] | 1.004  [0.999, 1.009] | 0.061 |
|  | Post 2^nd^ Wave |  | 6.22 | 6.18  [6.13, 6.23] | 0.04  [0.00, 0.08] | 1.006  [0.999, 1.012] | 0.050 |
|  | 3^rd^ Wave |  | 6.29 | 6.25  [6.20, 6.30] | 0.04  [0.00, 0.08] | 1.006  [0.999, 1.013] | 0.042 |

# Supplementary table S4: results by secondary outcome measures

| **Cohort** | **Period** | **Outcome** | **Observed*** | **Expected*** | **Difference**  **[95% CI]** | **Ratio**  **[95% CI]** | **P value** |
| --- | --- | --- | --- | --- | --- | --- | --- |
| Primary care | 1^st^ Wave | Depression presentations only | 0.44 | 0.79  [0.78, 0.80] | -0.35  [-0.36, -0.33] | 0.563  [0.55, 0.58] | <0.001 |
|  | Post 1^st^ Wave |  | 0.59 | 0.80  [0.79, 0.81] | -0.21  [-0.22, -0.19] | 0.740  [0.726, 0.754] | <0.001 |
|  | 2^nd^ Wave |  | 0.67 | 0.84  [0.82, 0.85] | -0.17  [-0.19, -0.15] | 0.798  [0.781, 0.816] | <0.001 |
|  | Post 2^nd^ Wave |  | 0.70 | 0.84  [0.82, 0.86] | -0.14  [-0.16, -0.11] | 0.838  [0.816, 0.861] | <0.001 |
|  | 3^rd^ Wave |  | 0.69 | 0.84  [0.82, 0.87] | -0.16  [-0.18, -0.13] | 0.814  [0.791, 0.839] | <0.001 |
|  | 1^st^ Wave | Anxiety presentations only | 0.43 | 0.68  [0.67, 0.69] | -0.25  [-0.26, -0.24] | 0.636  [0.62, 0.65] | <0.001 |
|  | Post 1^st^ Wave |  | 0.53 | 0.70  [0.69, 0.71] | -0.17  [-0.18, -0.16] | 0.760  [0.747, 0.774] | <0.001 |
|  | 2^nd^ Wave |  | 0.59 | 0.72  [0.72, 0.73] | -0.13  [-0.14, -0.12] | 0.820  [0.807, 0.834] | <0.001 |
|  | Post 2^nd^ Wave |  | 0.62 | 0.74  [0.73, 0.75] | -0.12  [-0.13, -0.10] | 0.841  [0.824, 0.858] | <0.001 |
|  | 3^rd^ Wave |  | 0.60 | 0.75  [0.74, 0.76] | -0.15  [-0.16, -0.13] | 0.806  [0.791, 0.821] | <0.001 |
|  | 1^st^ Wave | Medication of antidepressants | 5.21 | 5.55  [5.51, 5.59] | -0.34  [-0.36, -0.32] | 0.939  [0.935, 0.943] | <0.001 |
|  | Post 1^st^ Wave |  | 5.56 | 5.60  [5.56, 5.64] | -0.04  [-0.07, -0.02] | 0.992  [0.988, 0.996] | <0.001 |
|  | 2^nd^ Wave |  | 5.78 | 5.76  [5.72, 5.80] | 0.03  [0.00, 0.06] | 1.005  [0.999, 1.010] | 0.056 |
|  | Post 2^nd^ Wave |  | 5.97 | 5.93  [5.88, 5.98] | 0.04  [0.00, 0.08] | 1.007  [1.000, 1.013] | 0.037 |
|  | 3^rd^ Wave |  | 6.04 | 5.99  [5.94, 6.05] | 0.04  [0.00, 0.08] | 1.007  [1.000, 1.014] | 0.034 |
|  | 1^st^ Wave | Medication of anxiolytics | 0.73 | 0.76  [0.74, 0.77] | -0.03  [-0.04, -0.02] | 0.960  [0.948, 0.972] | <0.001 |
|  | Post 1^st^ Wave |  | 0.75 | 0.77  [0.75, 0.78] | -0.02  [-0.03, -0.01] | 0. 973  [0.961, 0.984] | <0.001 |
|  | 2^nd^ Wave |  | 0.77 | 0.76  [0.75, 0.78] | 0.00  [-0.01, 0.01] | 1.004  [0.990, 1.018] | 0.299 |
|  | Post 2^nd^ Wave |  | 0.75 | 0.76  [0.74, 0.78] | -0.01  [-0.02, 0.01] | 0.992  [0.974, 1.011] | 0.188 |
|  | 3^rd^ Wave |  | 0.75 | 0.76  [0.74, 0.78] | -0.01  [-0.02, 0.01] | 0.993  [0.972, 1.013] | 0.204 |

# Supplementary Figure S1: plots by secondary outcome measures


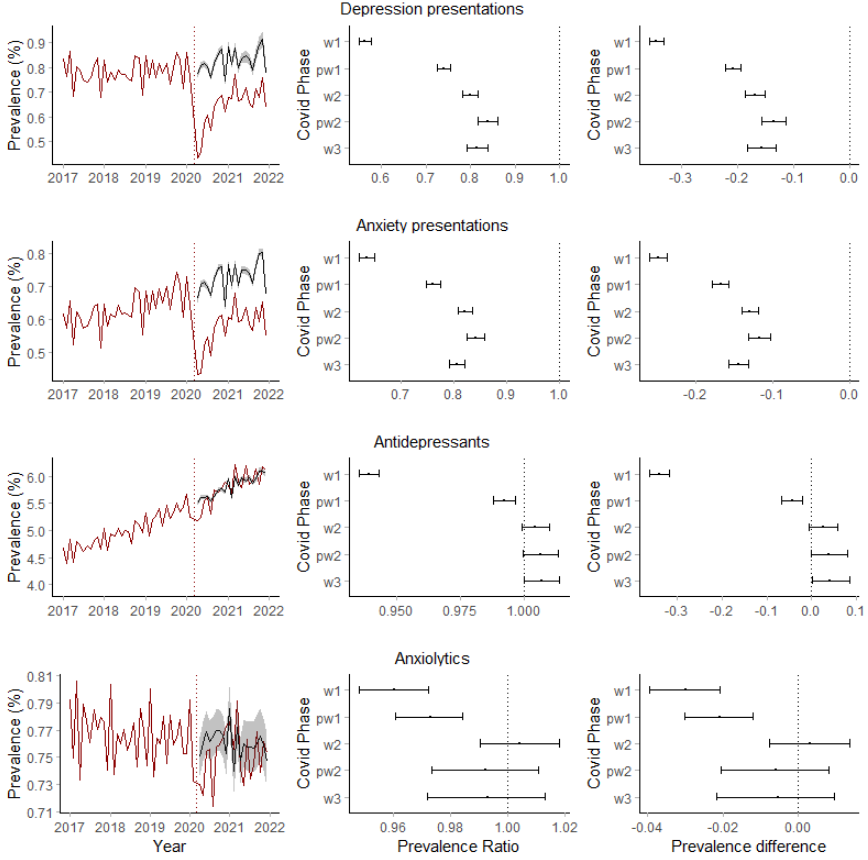


# Supplementary table S5: results by gender

| **Cohort** | **Outcome** | **Subgroup** | **Period** | **Observed*** | **Expected*** | **Difference**  **[95% CI]** | **Ratio**  **[95% CI]** | **P-value** |
| --- | --- | --- | --- | --- | --- | --- | --- | --- |
| Survey | Psychological distress | Women | 1^st^ Wave | 34.71 | 23.56  [21.80, 25.24] | 11.15  [8.96, 13.01] | 1.473  [1.363, 1.590] | <0.001 |
|  |  |  | Post 1^st^ Wave | 28.07 | 22.65  [21.08, 24.25] | 5.41  [3.42, 7.28] | 1.239  [1.145, 1.342] | <0.001 |
|  |  |  | 2^nd^ Wave | 33.21 | 24.65  [22.59, 26.74] | 8.57  [6.31, 10.84] | 1.348  [1.242, 1.472] | <0.001 |
|  |  |  | Post 2^nd^ Wave | 29.23 | 24.21  [21.92, 26.78] | 5.02  [2.12, 7.80] | 1.208  [1.083, 1.352] | 0.002 |
|  |  |  | 3^rd^ Wave | 25.03 | 23.45  [20.57, 26.30] | 1.58  [-1.68, 4.71] | 1.068  [0.935, 1.227] | 0.171 |
|  |  | Men | 1^st^ Wave | 21.81 | 17.90  [16.33, 19.55] | 3.92  [1.80, 6.13] | 1.219  [1.093, 1.366] | 0.003 |
|  |  |  | Post 1^st^ Wave | 19.45 | 16.79  [16.27, 18.48] | 2.66  [0.48, 4.69] | 1.158  [1.027, 1.295] | 0.010 |
|  |  |  | 2^nd^ Wave | 21.79 | 18.61  [16.68, 20.90] | 3.18  [0.84, 5.37] | 1.171  [1.042, 1.315] | 0.013 |
|  |  |  | Post 2^nd^ Wave | 19.98 | 18.68  [16.37, 21.29] | 1.30  [-1.76, 4.09] | 1.069  [0.917, 1.243] | 0.206 |
|  |  |  | 3^rd^ Wave | 17.27 | 17.50  [14.80, 20.66] | -0.23  [-3.74, 2.80] | 0.987  [0.818, 1.181] | 0.503 |
| Primary care | Presentations for anxiety or depression | Women | 1^st^ Wave | 0.89 | 1.44  [1.42, 1.46] | -0.54  [-0.57, -0.52] | 0.621  [0.606, 0.635] | <0.001 |
|  |  |  | Post 1^st^ Wave | 1.12 | 1.45  [1.43, 1.47] | -0.33  [-0.36, -0.30] | 0.772  [0.757, 0.787] | <0.001 |
|  |  |  | 2^nd^ Wave | 1.25 | 1.49  [1.47, 1.52] | -0.24  [-0.28, -0.21] | 0.836  [0.818, 0.854] | <0.001 |
|  |  |  | Post 2^nd^ Wave | 1.29 | 1.51  [1.47, 1.55] | -0.21  [-0.25, -0.17] | 0.859  [0.835, 0.883] | <0.001 |
|  |  |  | 3^rd^ Wave | 1.25 | 1.50  [1.46, 1.55] | -0.25  [-0.30, -0.21] | 0.833  [0.807, 0.860] | <0.001 |
|  |  | Men | 1^st^ Wave | 0.46 | 0.84  [0.82, 0.86] | -0.38  [-0.40, -0.36] | 0.551  [0.535, 0.568] | <0.001 |
|  |  |  | Post 1^st^ Wave | 0.60 | 0.85  [0.83, 0.87] | -0.25  [-0.27, -0.23] | 0.703  [0.685, 0.722] | <0.001 |
|  |  |  | 2^nd^ Wave | 0.66 | 0.89  [0.86, 0.91] | -0.23  [-0.25, -0.20] | 0.741  [0.721, 0.764] | <0.001 |
|  |  |  | Post 2^nd^ Wave | 0.69 | 0.90  [0.87, 0.93] | -0.20  [-0.23, -0.17] | 0.773  [0.746, 0.805] | <0.001 |
|  |  |  | 3^rd^ Wave | 0.68 | 0.91  [0.87, 0.94] | -0.23  [-0.27, -0.19] | 0.748  [0.718, 0.783] | <0.001 |
|  | Medications for anxiety or depression | Women | 1^st^ Wave | 7.29 | 7.72  [7.66, 7.79] | -0.43  [-0.47, -0.39] | 0.945  [0.940, 0.949] | <0.001 |
|  |  |  | Post 1^st^ Wave | 7.77 | 7.80  [7.74, 7.87] | -0.03  [-0.08, 0.01] | 0.996  [0.990, 1.001] | 0.045 |
|  |  |  | 2^nd^ Wave | 8.09 | 7.99  [7.92, 8.06] | 0.10  [0.05, 0.15] | 1.013  [1.006, 1.019] | <0.001 |
|  |  |  | Post 2^nd^ Wave | 8.33 | 8.21  [8.14, 8.29] | 0.12  [0.05, 0.18] | 1.015  [1.006, 1.022] | <0.001 |
|  |  |  | 3^rd^ Wave | 8.43 | 8.30  [8.22, 8.38] | 0.13  [0.06, 0.20] | 1.016  [1.008, 1.024] | <0.001 |
|  |  | Men | 1^st^ Wave | 3.61 | 3.89  [3.85, 3.94] | -0.28  [-0.31, -0.25] | 0.929  [0.922, 0.935] | <0.001 |
|  |  |  | Post 1^st^ Wave | 3.84 | 3.93  [3.88, 3.97] | -0.09  [-0.12, -0.06] | 0.977  [0.969, 0.985] | <0.001 |
|  |  |  | 2^nd^ Wave | 3.99 | 4.04  [3.99, 4.09] | -0.06  [-0.09, -0.02] | 0.986  [0.977, 0.996] | 0.004 |
|  |  |  | Post 2^nd^ Wave | 4.11 | 4.16  [4.10, 4.22] | -0.05  [-0.10, 0.00] | 0.988  [0.976, 0.999] | 0.014 |
|  |  |  | 3^rd^ Wave | 4.15 | 4.20  [4.14, 4.26] | -0.05  [-0.10, 0.00] | 0.987  [0.976, 0.999] | 0.020 |

# Supplementary Figure S2: time-series plots by gender


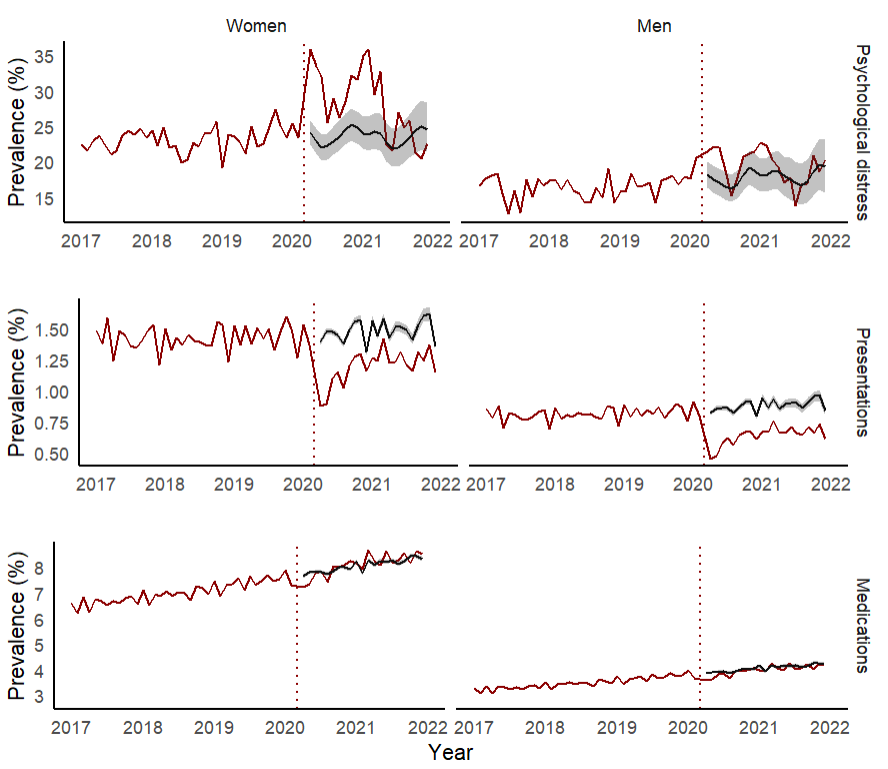


# Supplementary table S6: results by age-group

| **Subgroup** | **Cohort** | **Outcome** | **Period** | **Observed*** | **Expected*** | **Difference**  **[95% CI]** | **Ratio**  **[95% CI]** | **P-value** |
| --- | --- | --- | --- | --- | --- | --- | --- | --- |
| 16-24 | Survey | Psychological distress | 1^st^ Wave | 38.47 | 32.75  [28.66, 37.06] | 5.71  [0.70, 10.51] | 1.174  [1.019, 1.359] | 0.030 |
|  |  |  | Post 1^st^ Wave | 28.15 | 27.18  [23.28, 31.26] | 0.97  [-3.97, 5.76] | 1.036  [0.872, 1.243] | 0.337 |
|  |  |  | 2^nd^ Wave | 35.58 | 32.40  [27.44, 37.81] | 3.18  [-3.12, 8.98] | 1.098  [0.915, 1.315] | 0.141 |
|  |  |  | Post 2^nd^ Wave | 33.57 | 34.66  [28.29, 41.73] | -1.09  [-9.12, 6.69] | 0.969  [0.764, 1.225] | 0.566 |
|  |  |  | 3^rd^ Wave | 24.89 | 30.18  [22.94, 37.97] | -5.29  [-13.91, 2.55] | 0.825  [0.621, 1.106] | 0.928 |
| 25-34 |  |  | 1^st^ Wave | 36.85 | 25.13  [21.43, 29.04] | 11.72  [6.98, 15.97] | 1.467  [1.245, 1.728] | <0.001 |
|  |  |  | Post 1^st^ Wave | 30.78 | 23.17  [19.70, 26.79] | 7.60  [2.71, 12.07] | 1.328  [1.106, 1.583] | 0.008 |
|  |  |  | 2^nd^ Wave | 32.34 | 27.08  [22.30, 32.21] | 5.25  [-0.27, 10.70] | 1.194  [0.991, 1.475] | 0.061 |
|  |  |  | Post 2^nd^ Wave | 29.12 | 26.40  [20.81, 32.51] | 2.73  [-4.65, 9.69] | 1.103  [0.848, 1.439] | 0.213 |
|  |  |  | 3^rd^ Wave | 26.16 | 26.65  [19.54, 34.65] | -0.49  [-9.04, 7.38] | 0.982  [0.727, 1.350] | 0.478 |
| 35-49 |  |  | 1^st^ Wave | 31.34 | 22.93  [20.56, 25.49] | 8.41  [5.41, 11.36] | 1.366  [1.220, 1.534] | <0.001 |
|  |  |  | Post 1^st^ Wave | 26.71 | 23.24  [20.72, 25.73] | 3.47  [0.63, 6.41] | 1.149  [1.025, 1.302] | 0.025 |
|  |  |  | 2^nd^ Wave | 31.60 | 25.49  [22.51, 28.78] | 6.11  [2.31, 9.51] | 1.240  [1.080, 1.411] | 0.005 |
|  |  |  | Post 2^nd^ Wave | 26.09 | 25.36  [21.90, 29.47] | 0.74  [-3.81, 4.91] | 1.029  [0.864, 1.225] | 0.361 |
|  |  |  | 3^rd^ Wave | 24.22 | 25.46  [20.75, 30.35] | -1.24  [-6.70, 3.55] | 0.951  [0.772, 1.164] | 0.662 |
| 50-64 |  |  | 1^st^ Wave | 26.61 | 19.33  [17.09, 21.35] | 7.28  [4.93, 9.79] | 1.377  [1.236, 1.554] | <0.001 |
|  |  |  | Post 1^st^ Wave | 23.50 | 20.13  [17.98, 22.24] | 3.37  [0.90, 5.88] | 1.167  [1.041, 1.319] | 0.010 |
|  |  |  | 2^nd^ Wave | 26.44 | 21.17  [18.53, 23.78] | 5.27  [2.32, 8.29] | 1.249  [1.098, 1.433] | 0.005 |
|  |  |  | Post 2^nd^ Wave | 23.64 | 20.00  [17.05, 23.22] | 3.63  [0.07, 7.19] | 1.182  [1.003, 1.405] | 0.052 |
|  |  |  | 3^rd^ Wave | 20.73 | 20.05  [16.41, 23.87] | 0.68  [-3.55, 4.85] | 1.034  [0.853, 1.288] | 0.312 |
| 65+ |  |  | 1^st^ Wave | 18.56 | 12.36  [10.56, 14.19] | 6.20  [3.55, 8.52] | 1.501  [1.261, 1.785] | <0.001 |
|  |  |  | Post 1^st^ Wave | 16.31 | 12.08  [10.36, 13.88] | 4.23  [2.19, 6.36] | 1.350  [1.164, 1.576] | <0.001 |
|  |  |  | 2^nd^ Wave | 20.13 | 12.09  [10.20, 14.12] | 8.04  [5.65, 10.27] | 1.665  [1.415, 1.986] | <0.001 |
|  |  |  | Post 2^nd^ Wave | 18.82 | 11.82  [9.37, 14.53] | 6.99  [3.39, 10.29] | 1.591  [1.240, 2.046] | 0.003 |
|  |  |  | 3^rd^ Wave | 15.93 | 10.80  [8.18, 13.65] | 5.13  [1.95, 8.30] | 1.475  [1.142, 1.974] | 0.017 |
| 16-24 | Primary care | Presentations for anxiety or depression | 1^st^ Wave | 0.90 | 1.68  [1.63, 1.73] | -0.78  [-0.83, -0.73] | 0.534  [0.512, 0.559] | <0.001 |
|  |  |  | Post 1^st^ Wave | 1.14 | 1.50  [1.45, 1.55] | -0.36  [-0.42, -0.31] | 0.758  [0.727, 0.789] | <0.001 |
|  |  |  | 2^nd^ Wave | 1.41 | 1.74  [1.68, 1.81] | -0.33  [-0.40, -0.26] | 0.812  [0.776, 0.846] | <0.001 |
|  |  |  | Post 2^nd^ Wave | 1.57 | 1.83  [1.74, 1.93] | -0.27  [-0.37, -0.17] | 0.855  [0.808, 0.904] | <0.001 |
|  |  |  | 3^rd^ Wave | 1.38 | 1.69  [1.59, 1.80] | -0.31  [-0.42, -0.20] | 0.817  [0.764, 0.872] | <0.001 |
| 25-34 |  |  | 1^st^ Wave | 0.88 | 1.44  [1.40, 1.48] | -0.56  [-0.60, -0.52] | 0.614  [0.591, 0.638] | <0.001 |
|  |  |  | Post 1^st^ Wave | 1.16 | 1.53  [1.49, 1.58] | -0.37  [-0.42, -0.32] | 0.759  [0.734, 0.784] | <0.001 |
|  |  |  | 2^nd^ Wave | 1.26 | 1.55  [1.50, 1.60] | -0.28  [-0.34, -0.23] | 0.816  [0.786, 0.848] | <0.001 |
|  |  |  | Post 2^nd^ Wave | 1.30 | 1.56  [1.49, 1.63] | -0.26  [-0.33, -0.18] | 0.836  [0.797, 0.877] | <0.001 |
|  |  |  | 3^rd^ Wave | 1.28 | 1.63  [1.55, 1.72] | -0.35  [-0.44, -0.27] | 0.785  [0.743, 0.828] | <0.001 |
| 35-49 |  |  | 1^st^ Wave | 0.75 | 1.21  [1.18, 1.24] | -0.46  [-0.49, -0.43] | 0.620  [0.598, 0.641] | <0.001 |
|  |  |  | Post 1^st^ Wave | 0.94 | 1.25  [1.22, 1.28] | -0.31  [-0.35, -0.28] | 0.749  [0.724, 0.772] | <0.001 |
|  |  |  | 2^nd^ Wave | 1.02 | 1.28  [1.24, 1.32] | -0.26  [-0.30, -0.22] | 0.796  [0.769, 0.822] | <0.001 |
|  |  |  | Post 2^nd^ Wave | 1.04 | 1.27  [1.23, 1.32] | -0.23  [-0.29, -0.18] | 0.818  [0.783, 0.852] | <0.001 |
|  |  |  | 3^rd^ Wave | 1.03 | 1.29  [1.24, 1.36] | -0.27  [-0.33, -0.21] | 0.793  [0.756, 0.831] | <0.001 |
| 50-64 |  |  | 1^st^ Wave | 0.59 | 0.97  [0.95, 1.00] | -0.38  [-0.41, -0.35] | 0.609  [0.585, 0.635] | <0.001 |
|  |  |  | Post 1^st^ Wave | 0.74 | 1.02  [0.99, 1.05] | -0.27  [-0.30, -0.24] | 0.732  [0.706, 0.758] | <0.001 |
|  |  |  | 2^nd^ Wave | 0.80 | 1.01  [0.97, 1.04] | -0.21  [-0.25, -0.17] | 0.795  [0.763, 0.828] | <0.001 |
|  |  |  | Post 2^nd^ Wave | 0.81 | 0.99  [0.95, 1.04] | -0.18  [-0.23, -0.14] | 0.815  [0.775, 0.855] | <0.001 |
|  |  |  | 3^rd^ Wave | 0.82 | 1.01  [0.95, 1.07] | -0.19  [-0.25, -0.13] | 0.814  [0.768, 0.864] | <0.001 |
| 65+ |  |  | 1^st^ Wave | 0.32 | 0.57  [0.55, 0.59] | -0.25  [-0.27, -0.22] | 0.566  [0.536, 0.601] | <0.001 |
|  |  |  | Post 1^st^ Wave | 0.44 | 0.59  [0.57, 0.61] | -0.15  [-0.17, -0.12] | 0.747  [0.712, 0.785] | <0.001 |
|  |  |  | 2^nd^ Wave | 0.44 | 0.57  [0.55, 0.60] | -0.13  [-0.16, -0.10] | 0.772  [0.734, 0.817] | <0.001 |
|  |  |  | Post 2^nd^ Wave | 0.45 | 0.58  [0.54, 0.61] | -0.13  [-0.16, -0.09] | 0.779  [0.729, 0.839] | <0.001 |
|  |  |  | 3^rd^ Wave | 0.48 | 0.58  [0.54, 0.62] | -0.10  [-0.14, -0.06] | 0.830  [0.771, 0.894] | <0.001 |
| 16-24 |  | Medications for anxiety or depression | 1^st^ Wave | 2.78 | 3.18  [3.11, 3.26] | -0.39  [-0.46, -0.34] | 0.876  [0.858, 0.894] | <0.001 |
|  |  |  | Post 1^st^ Wave | 2.84 | 2.89  [2.82, 2.97] | -0.05  [-0.12, 0.02] | 0.983  [0.959, 1.007] | 0.074 |
|  |  |  | 2^nd^ Wave | 3.20 | 3.20  [3.11, 3.28] | 0.00  [-0.08, 0.08] | 1.000  [0.974, 1.026] | 0.478 |
|  |  |  | Post 2^nd^ Wave | 3.59 | 3.49  [3.39, 3.60] | 0.10  [-0.01, 0.20] | 1.029  [0.998, 1.057] | 0.051 |
|  |  |  | 3^rd^ Wave | 3.28 | 3.23  [3.13, 3.35] | 0.05  [-0.06, 0.15] | 1.015  [0.982, 1.048] | 0.236 |
| 25-34 |  |  | 1^st^ Wave | 4.09 | 4.44  [4.37, 4.53] | -0.35  [-0.41, -0.29] | 0.921  [0.908, 0.935] | <0.001 |
|  |  |  | Post 1^st^ Wave | 4.43 | 4.46  [4.38, 4.57] | -0.03  [-0.11, 0.05] | 0.993  [0.976, 1.010] | 0.257 |
|  |  |  | 2^nd^ Wave | 4.74 | 4.58  [4.50, 4.70] | 0.15  [0.06, 0.23] | 1.033  [1.014, 1.052] | <0.001 |
|  |  |  | Post 2^nd^ Wave | 4.94 | 4.70  [4.61, 4.83] | 0.24  [0.13, 0.34] | 1.051  [1.026, 1.074] | <0.001 |
|  |  |  | 3^rd^ Wave | 4.93 | 4.72  [4.61, 4.87] | 0.20  [0.06, 0.32] | 1.043  [1.013, 1.069] | 0.003 |
| 35-49 |  |  | 1^st^ Wave | 5.83 | 6.20  [6.12, 6.28] | -0.37  [-0.42, -0.33] | 0.940  [0.933, 0.947] | <0.001 |
|  |  |  | Post 1^st^ Wave | 6.10 | 6.14  [6.06, 6.22] | -0.04  [-0.10, 0.02] | 0.994  [0.983, 1.002] | 0.075 |
|  |  |  | 2^nd^ Wave | 6.38 | 6.33  [6.25, 6.42] | 0.06  [-0.01, 0.12] | 1.009  [0.998, 1.019] | 0.050 |
|  |  |  | Post 2^nd^ Wave | 6.58 | 6.50  [6.40, 6.59] | 0.09  [0.01, 0.16] | 1.013  [1.002, 1.025] | 0.022 |
|  |  |  | 3^rd^ Wave | 6.54 | 6.46  [6.36, 6.56] | 0.09  [0.00, 0.17] | 1.013  [1.001, 1.027] | 0.030 |
| 50-64 |  |  | 1^st^ Wave | 7.18 | 7.60  [7.50, 7.69] | -0.42  [-0.47, -0.36] | 0.945  [0.938, 0.952] | <0.001 |
|  |  |  | Post 1^st^ Wave | 7.61 | 7.76  [7.64, 7.85] | -0.14  [-0.21, -0.07] | 0.982  [0.973, 0.991] | <0.001 |
|  |  |  | 2^nd^ Wave | 7.85 | 7.94  [7.81, 8.03] | -0.09  [-0.16, -0.01] | 0.989  [0.980, 0.999] | 0.016 |
|  |  |  | Post 2^nd^ Wave | 7.99 | 8.12  [7.98, 8.22] | -0.13  [-0.22, -0.04] | 0.984  [0.973, 0.995] | 0.004 |
|  |  |  | 3^rd^ Wave | 8.22 | 8.31  [8.16, 8.43] | -0.09  [-0.19, 0.02] | 0.989  [0.977, 1.002] | 0.061 |
| 65+ |  |  | 1^st^ Wave | 6.23 | 6.52  [6.43, 6.62] | -0.29  [-0.34, -0.24] | 0.955  [0.948, 0.962] | <0.001 |
|  |  |  | Post 1^st^ Wave | 6.57 | 6.66  [6.56, 6.77] | -0.09  [-0.15, -0.02] | 0.986  [0.977, 0.997] | 0.003 |
|  |  |  | 2^nd^ Wave | 6.70 | 6.77  [6.65, 6.88] | -0.06  [-0.14, 0.02] | 0.991  [0.980, 1.003] | 0.072 |
|  |  |  | Post 2^nd^ Wave | 6.78 | 6.92  [6.79, 7.04] | -0.14  [-0.23, -0.03] | 0.980  [0.967, 0.995] | 0.007 |
|  |  |  | 3^rd^ Wave | 7.04 | 7.07  [6.92, 7.21] | -0.03  [-0.14, 0.09] | 0.996  [0.981, 1.014] | 0.344 |

# Supplementary Figure S3: time-series plots by age-group


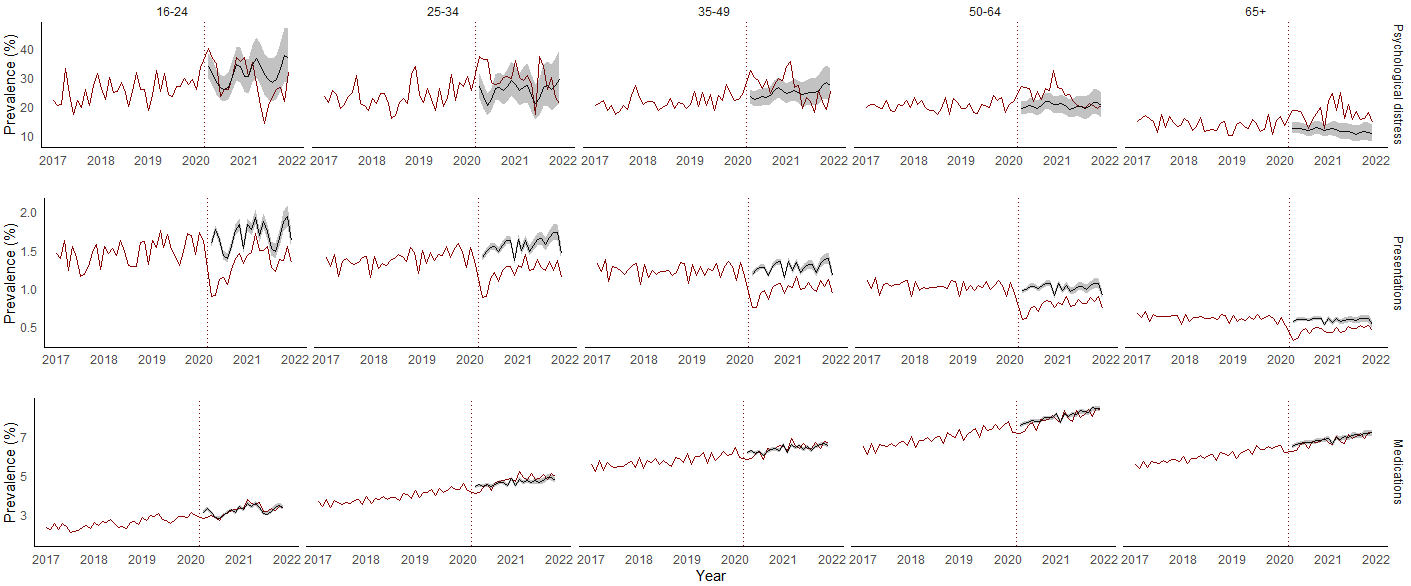


# Supplementary table S7: results by ethnicity

| **Subgroup** | **Cohort** | **Outcome** | **Period** | **Observed*** | **Expected*** | **Difference**  **[95% CI]** | Ratio  [95% CI] | p-value |
| --- | --- | --- | --- | --- | --- | --- | --- | --- |
| Asian | Survey | Psychological distress | 1^st^ Wave | 27.24 | 24.59  [20.41, 29.12] | 2.64  [-3.54, 8.28] | 1.108  [0.870, 1.383] | 0.167 |
|  |  |  | Post 1^st^ Wave | 25.22 | 26.11  [21.81, 30.54] | -0.89  [-6.50, 5.49] | 0.966  [0.775, 1.203] | 0.574 |
|  |  |  | 2^nd^ Wave | 26.49 | 26.74  [21.70, 32.37] | -0.25  [-6.70, 5.83] | 0.991  [0.783, 1.263] | 0.46 |
|  |  |  | Post 2^nd^ Wave | 26.23 | 30.41  [23.30, 38.21] | -4.18  [-12.66, 4.58] | 0.863  [0.649, 1.192] | 0.817 |
|  |  |  | 3^rd^ Wave | 20.04 | 31.82  [23.30, 41.16] | -11.78  [-21.64, -2.90] | 0.630  [0.459, 0.880] | <0.001 |
| Black |  |  | 1^st^ Wave | 31.31 | 27.17  [19.78, 35.37] | 4.14  [-7.73, 15.96] | 1.152  [0.754, 1.717] | 0.241 |
|  |  |  | Post 1^st^ Wave | 24.24 | 24.55  [18.01, 31.64] | -0.31  [-9.87, 8.40] | 0.988  [0.667, 1.402] | 0.467 |
|  |  |  | 2^nd^ Wave | 28.09 | 24.68  [17.39, 32.95] | 3.42  [-7.20, 12.63] | 1.138  [0.759, 1.656] | 0.242 |
|  |  |  | Post 2^nd^ Wave | 21.53 | 29.38  [19.26, 40.93] | -7.85  [-19.79, 3.83] | 0.733  [0.480, 1.200] | 0.95 |
|  |  |  | 3^rd^ Wave | 25.47 | 26.91  [15.73, 40.81] | -1.44  [-17.77, 11.15] | 0.946  [0.543, 1.673] | 0.467 |
| White |  |  | 1^st^ Wave | 28.47 | 20.40  [19.10, 21.67] | 8.07  [6.57, 9.65] | 1.395  [1.308, 1.496] | <0.001 |
|  |  |  | Post 1^st^ Wave | 23.71 | 19.35  [18.18, 20.52] | 4.35  [2.91, 5.82] | 1.225  [1.144, 1.319] | <0.001 |
|  |  |  | 2^nd^ Wave | 28.01 | 21.17  [19.68, 22.72] | 6.84  [5.09, 8.60] | 1.323  [1.229, 1.432] | <0.001 |
|  |  |  | Post 2^nd^ Wave | 24.84 | 20.60  [18.85, 22.51] | 4.24  [2.02, 6.33] | 1.206  [1.091, 1.330] | 0.002 |
|  |  |  | 3^rd^ Wave | 21.30 | 19.81  [17.71, 21.95] | 1.49  [-0.82, 3.85] | 1.075  [0.963, 1.215] | 0.108 |
| Other |  |  | 1^st^ Wave | 40.35 | 23.23  [16.47, 31.99] | 17.12  [5.44, 27.89] | 1.737  [1.184, 2.476] | 0.016 |
|  |  |  | Post 1^st^ Wave | 33.91 | 24.89  [7.08, 34.03] | 9.03  [-1.74, 19.58] | 1.363  [0.944, s.039] | 0.086 |
|  |  |  | 2^nd^ Wave | 29.72 | 33.48  [22.96, 45.30] | -3.76  [-16.53, 9.31] | 0.888  [0.615, 1.383] | 0.606 |
|  |  |  | Post 2^nd^ Wave | 28.68 | 38.48  [25.56, 53.60] | -9.80  [-27.14, 6.27] | 0.745  [0.466, 1.249] | 0.903 |
|  |  |  | 3^rd^ Wave | 27.53 | 29.04  [14.99, 47.33] | -1.51  [-20.17, 13.40] | 0.948  [0.561, 1.789] | 0.398 |
| Asian | Primary care | Presentations for anxiety or depression | 1^st^ Wave | 0.35 | 0.63  [0.59, 0.67] | -0.28  [-0.32, -0.23] | 0.557  [0.503, 0.611] | <0.001 |
|  |  |  | Post 1^st^ Wave | 0.47 | 0.64  [0.59, 0.68] | -0.17  [-0.21, -0.12] | 0.739  [0.683, 0.803] | <0.001 |
|  |  |  | 2^nd^ Wave | 0.51 | 0.66  [0.60, 0.71] | -0.14  [-0.20, -0.09] | 0.780  [0.716, 0.854] | <0.001 |
|  |  |  | Post 2^nd^ Wave | 0.54 | 0.68  [0.61, 0.74] | -0.13  [-0.21, -0.06] | 0.803  [0.719, 0.902] | <0.001 |
|  |  |  | 3^rd^ Wave | 0.53 | 0.68  [0.60, 0.77] | -0.15  [-0.23, -0.06] | 0.780  [0.692, 0.893] | <0.001 |
| Black |  |  | 1^st^ Wave | 0.39 | 0.66  [0.61, 0.71] | -0.27  [-0.32, -0.21] | 0.598  [0.534, 0.670] | <0.001 |
|  |  |  | Post 1^st^ Wave | 0.49 | 0.66  [0.60, 0.72] | -0.18  [-0.24, -0.12] | 0.733  [0.664, 0.809] | <0.001 |
|  |  |  | 2^nd^ Wave | 0.53 | 0.67  [0.60, 0.73] | -0.13  [-0.21, -0.06] | 0.798  [0.715, 0.897] | <0.001 |
|  |  |  | Post 2^nd^ Wave | 0.59 | 0.70  [0.62, 0.79] | -0.12  [-0.21, -0.02] | 0.832  [0.728, 0.967] | 0.011 |
|  |  |  | 3^rd^ Wave | 0.58 | 0.70  [0.59, 0.80] | -0.12  [-0.23, -0.02] | 0.829  [0.711, 0.970] | 0.010 |
| White |  |  | 1^st^ Wave | 0.77 | 1.29  [1.28, 1.31] | -0.52  [-0.54, -0.50] | 0.600  [0.587, 0.611] | <0.001 |
|  |  |  | Post 1^st^ Wave | 0.98 | 1.31  [1.29, 1.33] | -0.33  [-0.35, -0.31] | 0.747  [0.735, 0.759] | <0.001 |
|  |  |  | 2^nd^ Wave | 1.09 | 1.36  [1.34, 1.38] | -0.27  [-0.30, -0.25] | 0.800  [0.785, 0.815] | <0.001 |
|  |  |  | Post 2^nd^ Wave | 1.12 | 1.37  [1.34, 1.40] | -0.24  [-0.28, -0.21] | 0.822  [0.801, 0.842] | <0.001 |
|  |  |  | 3^rd^ Wave | 1.09 | 1.38  [1.34, 1.41] | -0.28  [-0.32, -0.25] | 0.794  [0.772, 0.815] | <0.001 |
| Other |  |  | 1^st^ Wave | 0.47 | 0.80  [0.74, 0.86] | -0.33  [-0.40, -0.28] | 0.582  [0.524, 0.641] | <0.001 |
|  |  |  | Post 1^st^ Wave | 0.59 | 0.78  [0.72, 0.85] | -0.19  [-0.26, -0.12] | 0.762  [0.691, 0.832] | <0.001 |
|  |  |  | 2^nd^ Wave | 0.67 | 0.81  [0.73, 0.90] | -0.14  [-0.22, -0.06] | 0.828  [0.747, 0.922] | 0.001 |
|  |  |  | Post 2^nd^ Wave | 0.74 | 0.84  [0.74, 0.95] | -0.10  [-0.21, 0.0] | 0.884  [0.775, 1.003] | 0.026 |
|  |  |  | 3^rd^ Wave | 0.70 | 0.81  [0.70, 0.95] | -0.11  [-0.24, 0.00] | 0.864  [0.742, 1.007] | 0.030 |
| Asian |  | Medications for anxiety or depression | 1^st^ Wave | 2.06 | 2.25  [2.18, 2.34] | -0.19  [-0.25, -0.14] | 0.916  [0.889, 0.936] | <0.001 |
|  |  |  | Post 1^st^ Wave | 2.24 | 2.29  [2.22, 2.39] | -0.05  [-0.12, 0.00] | 0.977  [0.949, 0.999] | 0.020 |
|  |  |  | 2^nd^ Wave | 2.33 | 2.34  [2.26, 2.44] | -0.01  [-0.09, 0.04] | 0.994  [0.963, 1.019] | 0.266 |
|  |  |  | Post 2^nd^ Wave | 2.38 | 2.40  [2.30, 2.50] | -0.01  [-0.10, 0.06] | 0.994  [0.958, 1.028] | 0.308 |
|  |  |  | 3^rd^ Wave | 2.40 | 2.43  [2.33, 2.54] | -0.03  [-0.12, 0.05] | 0.987  [0.951, 1.021] | 0.166 |
| Black |  |  | 1^st^ Wave | 1.78 | 1.94  [1.84, 2.06] | -0.17  [-0.25, -0.10] | 0.915  [0.873, 0.947] | <0.001 |
|  |  |  | Post 1^st^ Wave | 1.88 | 1.95  [1.84, 2.06] | -0.06  [-0.16, 0.00] | 0.967  [0.923, 1.001] | 0.033 |
|  |  |  | 2^nd^ Wave | 1.97 | 1.98  [1.87, 2.10] | -0.01  [-0.11, 0.07] | 0.996  [0.947, 1.037] | 0.349 |
|  |  |  | Post 2^nd^ Wave | 2.06 | 2.06  [1.94, 2.20] | 0.00  [-0.12, 0.11] | 1.001  [0.945, 1.058] | 0.459 |
|  |  |  | 3^rd^ Wave | 2.09 | 2.06  [1.93, 2.20] | 0.03  [-0.10, 0.14] | 1.014  [0.956, 1.069] | 0.347 |
| White |  |  | 1^st^ Wave | 6.57 | 6.96  [6.92, 7.01] | -0.39  [-0.42, -0.37] | 0.943  [0.939, 0.947] | <0.001 |
|  |  |  | Post 1^st^ Wave | 6.98 | 7.03  [6.99, 7.09] | -0.05  [-0.09, -0.02] | 0.992  [0.987, 0.997] | <0.001 |
|  |  |  | 2^nd^ Wave | 7.26 | 7.23  [7.17, 7.28] | 0.03  [-0.01, 0.07] | 1.005  [0.999, 1.010] | 0.056 |
|  |  |  | Post 2^nd^ Wave | 7.47 | 7.43  [7.37, 7.50] | 0.04  [-0.01, 0.09] | 1.005  [0.998, 1.013] | 0.072 |
|  |  |  | 3^rd^ Wave | 7.59 | 7.53  [7.47, 7.61] | 0.05  [0.00, 0.12] | 1.007  [1.000, 1.016] | 0.040 |
| Other |  |  | 1^st^ Wave | 2.25 | 2.48  [2.38, 2.62] | -0.22  [-0.32, -0.16] | 0.909  [0.873, 0.935] | <0.001 |
|  |  |  | Post 1^st^ Wave | 2.45 | 2.47  [2.37, 2.61] | -0.02  [-0.12, 0.05] | 0.991  [0.951, 1.020] | 0.235 |
|  |  |  | 2^nd^ Wave | 2.61 | 2.53  [2.41, 2.65] | 0.08  [-0.03, 0.17] | 1.031  [0.990, 1.072] | 0.067 |
|  |  |  | Post 2^nd^ Wave | 2.76 | 2.62  [2.47, 2.75] | 0.14  [0.02, 0.28] | 1.052  [1.008, 1.110] | 0.017 |
|  |  |  | 3^rd^ Wave | 2.72 | 2.59  [2.43, 2.73] | 0.13  [0.01, 0.27] | 1.051  [1.005, 1.112] | 0.025 |

# Supplementary Figure S4: time-series plots by ethnicity


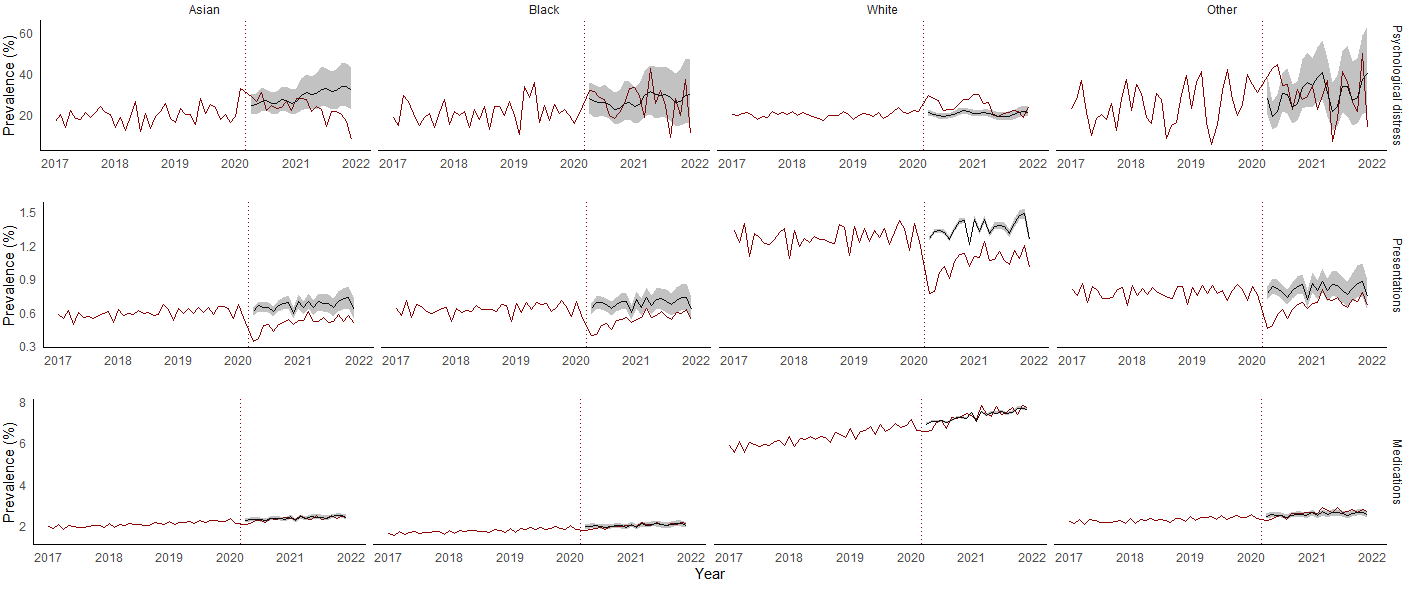


# Supplementary table S8: results by IMD quintile

| **Subgroup** | **Cohort** | **Outcome** | **Period** | **Observed*** | **Expected*** | **Difference**  **[95% CI]** | **Ratio**  **[95% CI]** | **p-value** |
| --- | --- | --- | --- | --- | --- | --- | --- | --- |
| 1 (lowest) | Survey | Psychological distress | 1^st^ Wave | 33.29 | 26.68  [23.61, 29.79] | 6.61  [2.70, 10.64] | 1.248  [1.093, 1.433] | 0.003 |
|  |  |  | Post 1^st^ Wave | 28.80 | 23.69  [20.90, 26.62] | 5.10  [1.55, 8.57] | 1.215  [1.060, 1.399] | 0.008 |
|  |  |  | 2^nd^ Wave | 32.95 | 29.03  [25.50, 32.90] | 3.93  [-0.43, 8.30] | 1.135  [0.986, 1.317] | 0.056 |
|  |  |  | Post 2^nd^ Wave | 28.47 | 27.90  [23.33, 32.41] | 0.57  [-4.87, 6.18] | 1.020  [0.849, 1.249] | 0.349 |
|  |  |  | 3^rd^ Wave | 28.43 | 27.56  [22.14, 33.24] | 0.87  [-5.59, 7.09] | 1.032  [0.829, 1.318] | 0.343 |
| 2 |  |  | 1^st^ Wave | 30.26 | 21.36  [18.55, 24.20] | 8.90  [5.13, 12.25] | 1.417  [1.218, 1.641] | <0.001 |
|  |  |  | Post 1^st^ Wave | 26.35 | 22.72  [19.77, 25.62] | 3.63  [-0.25, 7.55] | 1.160  [0.991, 1.370] | 0.045 |
|  |  |  | 2^nd^ Wave | 30.64 | 23.11  [19.75, 26.58] | 7.52  [3.64, 11.21] | 1.325  [1.142, 1.555] | 0.003 |
|  |  |  | Post 2^nd^ Wave | 25.27 | 21.66  [17.75, 25.51] | 3.61  [-0.88, 8.75] | 1.167  [0.964, 1.487] | 0.09 |
|  |  |  | 3^rd^ Wave | 24.23 | 23.40  [18.51, 28.74] | 0.83  [-5.03, 6.22] | 1.036  [0.819, 1.320] | 0.354 |
| 3 |  |  | 1^st^ Wave | 29.18 | 21.25  [18.76, 23.99] | 7.94  [4.88, 11.32] | 1.373  [1.209, 1.585] | 0.001 |
|  |  |  | Post 1^st^ Wave | 23.09 | 19.71  [17.21, 22.30] | 3.38  [0.59, 6.21] | 1.171  [1.026, 1.360] | 0.027 |
|  |  |  | 2^nd^ Wave | 28.31 | 21.96  [18.84, 25.41] | 6.35  [2.71, 10.13] | 1.289  [1.110, 1.536] | 0.006 |
|  |  |  | Post 2^nd^ Wave | 25.03 | 21.56  [18.07, 25.60] | 3.47  [-1.31, 7.99] | 1.161  [0.947, 1.430] | 0.100 |
|  |  |  | 3^rd^ Wave | 20.59 | 21.30  [16.89, 26.80] | -0.71  [-6.26, 4.18] | 0.967  [0.760, 1.244] | 0.561 |
| 4 |  |  | 1^st^ Wave | 26.31 | 18.18  [16.02, 20.44] | 8.12  [5.35, 11.01] | 1.447  [1.266, 1.669] | <0.001 |
|  |  |  | Post 1^st^ Wave | 22.42 | 16.91  [14.79, 19.26] | 5.52  [2.80, 8.19] | 1.326  [1.150, 1.542] | 0.001 |
|  |  |  | 2^nd^ Wave | 25.18 | 17.77  [15.10, 20.55] | 7.41  [4.46, 10.55] | 1.417  [1.221, 1.663] | <0.001 |
|  |  |  | Post 2^nd^ Wave | 25.22 | 18.84  [15.59, 22.43] | 6.38  [2.19, 10.76] | 1.339  [1.100, 1.675] | 0.018 |
|  |  |  | 3^rd^ Wave | 20.74 | 15.02  [11.62, 18.99] | 5.72  [1.44, 9.62] | 1.381  [1.079, 1.823] | 0.028 |
| 5 (highest) |  |  | 1^st^ Wave | 25.78 | 17.26  [14.93, 19.64] | 8.51  [5.79, 11.31] | 1.493  [1.299, 1.728] | <0.001 |
|  |  |  | Post 1^st^ Wave | 21.04 | 17.53  [15.33, 19.89] | 3.51  [0.65, 6.14] | 1.20  [1.033, 1.398] | 0.018 |
|  |  |  | 2^nd^ Wave | 24.41 | 18.26  [15.54, 20.99] | 6.15  [2.91, 9.45] | 1.337  [1.141, 1.592] | 0.003 |
|  |  |  | Post 2^nd^ Wave | 21.58 | 18.75  [15.22, 22.39] | 2.83  [-1.42, 6.77] | 1.151  [0.937, 1.431] | 0.089 |
|  |  |  | 3^rd^ Wave | 14.98 | 18.04  [13.91, 22.61] | -3.06  [-7.96, 1.49] | 0.830  [0.644, 1.100] | 0.926 |
| 1 (lowest) | Primary care | Presentations for anxiety or depression | 1^st^ Wave | 0.54 | 0.93  [0.90, 0.96] | -0.39  [-0.42, -0.36] | 0.580  [0.553, 0.607] | <0.001 |
|  |  |  | Post 1^st^ Wave | 0.70 | 0.93  [0.91, 0.97] | -0.24  [-0.27, -0.20] | 0.747  [0.718, 0.776] | <0.001 |
|  |  |  | 2^nd^ Wave | 0.78 | 0.97  [0.94, 1.01] | -0.19  [-0.23, -0.16] | 0.801  [0.766, 0.834] | <0.001 |
|  |  |  | Post 2^nd^ Wave | 0.82 | 0.97  [0.92, 1.02] | -0.15  [-0.20, -0.10] | 0.845  [0799, 0.890] | <0.001 |
|  |  |  | 3^rd^ Wave | 0.79 | 0.96  [0.91, 1.03] | -0.17  [-0.24, -0.11] | 0.824  [0.771, 0.875] | <0.001 |
| 2 |  |  | 1^st^ Wave | 0.60 | 1.04  [1.01, 1.07] | -0.44  [-0.47, -0.40] | 0.580  [0.556, 0.608] | <0.001 |
|  |  |  | Post 1^st^ Wave | 0.78 | 1.04  [1.01, 1.07] | -0.27  [-0.30, -0.23] | 0.745  [0.719, 0.775] | <0.001 |
|  |  |  | 2^nd^ Wave | 0.87 | 1.09  [1.05, 1.13] | -0.22  [-0.26, -0.18] | 0.800  [0.769, 0.835] | <0.001 |
|  |  |  | Post 2^nd^ Wave | 0.91 | 1.10  [1.05, 1.15] | -0.20  [-0.25, -0.14] | 0.821  [0.778, 0.867] | <0.001 |
|  |  |  | 3^rd^ Wave | 0.88 | 1.10  [1.04, 1.16] | -0.22  [-0.28, -0.16] | 0.800  [0.756, 0.850] | <0.001 |
| 3 |  |  | 1^st^ Wave | 0.66 | 1.10  [1.07, 1.13] | -0.44  [-0.48, -0.41] | 0.598  [0.573, 0.623] | <0.001 |
|  |  |  | Post 1^st^ Wave | 0.83 | 1.10  [1.07, 1.14] | -0.27  [-0.31, -0.24] | 0.753  [0.725, 0.779] | <0.001 |
|  |  |  | 2^nd^ Wave | 0.92 | 1.16  [1.12, 1.20] | -0.24  [-0.29, -0.20] | 0.793  [0.759, 0.824] | <0.001 |
|  |  |  | Post 2^nd^ Wave | 0.95 | 1.17  [1.12, 1.23] | -0.22  [-0.28, -0.16] | 0.816  [0.773, 0.857] | <0.001 |
|  |  |  | 3^rd^ Wave | 0.92 | 1.18  [1.12, 1.25] | -0.25  [-0.33, -0.19] | 0.783  [0.736, 0.829] | <0.001 |
| 4 |  |  | 1^st^ Wave | 0.69 | 1.17  [1.14, 1.20] | -0.48  [-0.51, -0.44] | 0.592  [0.570, 0.615] | <0.001 |
|  |  |  | Post 1^st^ Wave | 0.88 | 1.18  [1.15, 1.22] | -0.30  [-0.34, -0.26] | 0.746  [0.721, 0.772] | <0.001 |
|  |  |  | 2^nd^ Wave | 0.97 | 1.22  [1.18, 1.27] | -0.25  [-0.30, -0.21] | 0.793  [0.764, 0.825] | <0.001 |
|  |  |  | Post 2^nd^ Wave | 1.01 | 1.25  [1.19, 1.30] | -0.24  [-0.29, -0.18] | 0.811  [0.773, 0.848] | <0.001 |
|  |  |  | 3^rd^ Wave | 0.98 | 1.26  [1.19, 1.32] | -0.27  [-0.34, -0.20] | 0.783  [0.742, 0.829] | <0.001 |
| 5 (highest) |  |  | 1^st^ Wave | 0.91 | 1.47  [1.43, 1.50] | -0.56  [-0.60, -0.52] | 0.617  [0.596, 0.641] | <0.001 |
|  |  |  | Post 1^st^ Wave | 1.12 | 1.50  [1.46, 1.55] | -0.38  [-0.43, -0.34] | 0.745  [0.722, 0.769] | <0.001 |
|  |  |  | 2^nd^ Wave | 1.24 | 1.52  [1.47, 1.57] | -0.28  [-0.33, -0.23] | 0.816  [0.791, 0.846] | <0.001 |
|  |  |  | Post 2^nd^ Wave | 1.29 | 1.53  [1.47, 1.59] | -0.24  [-0.30, -0.18] | 0.844  [0.808, 0.881] | <0.001 |
|  |  |  | 3^rd^ Wave | 1.25 | 1.53  [1.46, 1.61] | -0.29  [-0.36, -0.21] | 0.814  [0.776, 0.857] | <0.001 |
| 1 (lowest) |  | Medications for anxiety or depression | 1^st^ Wave | 4.53 | 4.90  [4.83, 4.98] | -0.37  [-0.42, -0.32] | 0.924  [0.914, 0.934] | <0.001 |
|  |  |  | Post 1^st^ Wave | 4.87 | 4.93  [4.86, 5.02] | -0.06  [-0.12, -0.01] | 0.987  [0.977, 0.997] | 0.005 |
|  |  |  | 2^nd^ Wave | 5.09 | 5.09  [5.01, 5.18] | 0.01  [-0.06, 0.06] | 1.001  [0.99, 1.01] | 0.483 |
|  |  |  | Post 2^nd^ Wave | 5.26 | 5.25  [5.16, 5.34] | 0.01  [-0.07, 0.08] | 1.002  [0.987, 1.015] | 0.475 |
|  |  |  | 3^rd^ Wave | 5.36 | 5.31  [5.22, 5.41] | 0.06  [-0.04, 0.13] | 1.010  [0.993, 1.024] | 0.138 |
| 2 |  |  | 1^st^ Wave | 5.01 | 5.37  [5.28, 5.45] | -0.35  [-0.41, -0.31] | 0.934  [0.924, 0.942] | <0.001 |
|  |  |  | Post 1^st^ Wave | 5.35 | 5.40  [5.31, 5.48] | -0.05  [-0.11, 0.00] | 0.990  [0.980, 1.000] | 0.019 |
|  |  |  | 2^nd^ Wave | 5.58 | 5.55  [5.45, 5.63] | 0.03  [-0.04, 0.09] | 1.005  [0.993, 1.016] | 0.210 |
|  |  |  | Post 2^nd^ Wave | 5.73 | 5.72  [5.61, 5.81] | 0.01  [-0.07, 0.10] | 1.002  [0.988, 1.017] | 0.357 |
|  |  |  | 3^rd^ Wave | 5.84 | 5.78  [5.65, 5.87] | 0.07  [-0.02, 0.16] | 1.011  [0.996, 1.028] | 0.074 |
| 3 |  |  | 1^st^ Wave | 5.18 | 5.52  [5.44, 5.62] | -0.34  [-0.39, -0.29] | 0.939  [0.930, 0.947] | <0.001 |
|  |  |  | Post 1^st^ Wave | 5.51 | 5.57  [5.48, 5.67] | -0.06  [-0.12, 0.00] | 0.990  [0.978, 1.000] | 0.022 |
|  |  |  | 2^nd^ Wave | 5.74 | 5.72  [5.63, 5.83] | 0.01  [-0.06, 0.08] | 1.002  [0.990, 1.013] | 0.361 |
|  |  |  | Post 2^nd^ Wave | 5.90 | 5.89  [5.78, 6.00] | 0.01  [-0.07, 0.10] | 1.002  [0.988, 1.017] | 0.371 |
|  |  |  | 3^rd^ Wave | 5.99 | 5.96  [5.85, 6.09] | 0.03  [-0.06, 0.13] | 1.006  [0.991, 1.023] | 0.202 |
| 4 |  |  | 1^st^ Wave | 5.31 | 5.64  [5.56, 5.72] | -0.32  [-0.38, -0.28] | 0.943  [0.934, 0.951] | <0.001 |
|  |  |  | Post 1^st^ Wave | 5.66 | 5.69  [5.61, 5.77] | -0.03  [-0.09, 0.02] | 0.994  [0.984, 1.003] | 0.090 |
|  |  |  | 2^nd^ Wave | 5.88 | 5.83  [5.73, 5.92] | 0.05  [-0.02, 0.12] | 1.008  [0.996, 1.020] | 0.090 |
|  |  |  | Post 2^nd^ Wave | 6.05 | 5.97  [5.85, 6.07] | 0.08  [0.00, 0.18] | 1.014  [1.000, 1.029] | 0.031 |
|  |  |  | 3^rd^ Wave | 6.08 | 6.02  [5.90, 6.13] | 0.06  [-0.03, 0.16] | 1.010  [0.996, 1.027] | 0.096 |
| 5 (highest) |  |  | 1^st^ Wave | 7.33 | 7.71  [7.60, 7.82] | -0.38  [-0.44, -0.32] | 0.951  [0.942, 0.958] | <0.001 |
|  |  |  | Post 1^st^ Wave | 7.71 | 7.81  [7.70, 7.92] | -0.09  [-0.16, -0.03] | 0.988  [0.979, 0.997] | 0.002 |
|  |  |  | 2^nd^ Wave | 7.99 | 7.95  [7.83, 8.07] | 0.03  [-0.05, 0.11] | 1.004  [0.994, 1.015] | 0.195 |
|  |  |  | Post 2^nd^ Wave | 8.23 | 8.16  [8.02, 8.29] | 0.06  [-0.04, 0.18] | 1.008  [0.996, 1.022] | 0.092 |
|  |  |  | 3^rd^ Wave | 8.20 | 8.23  [8.10, 8.37] | -0.03  [-0.13, 0.08] | 0.996  [0.984, 1.010] | 0.325 |

# Supplementary Figure S5: time-series plots by IMD quintile


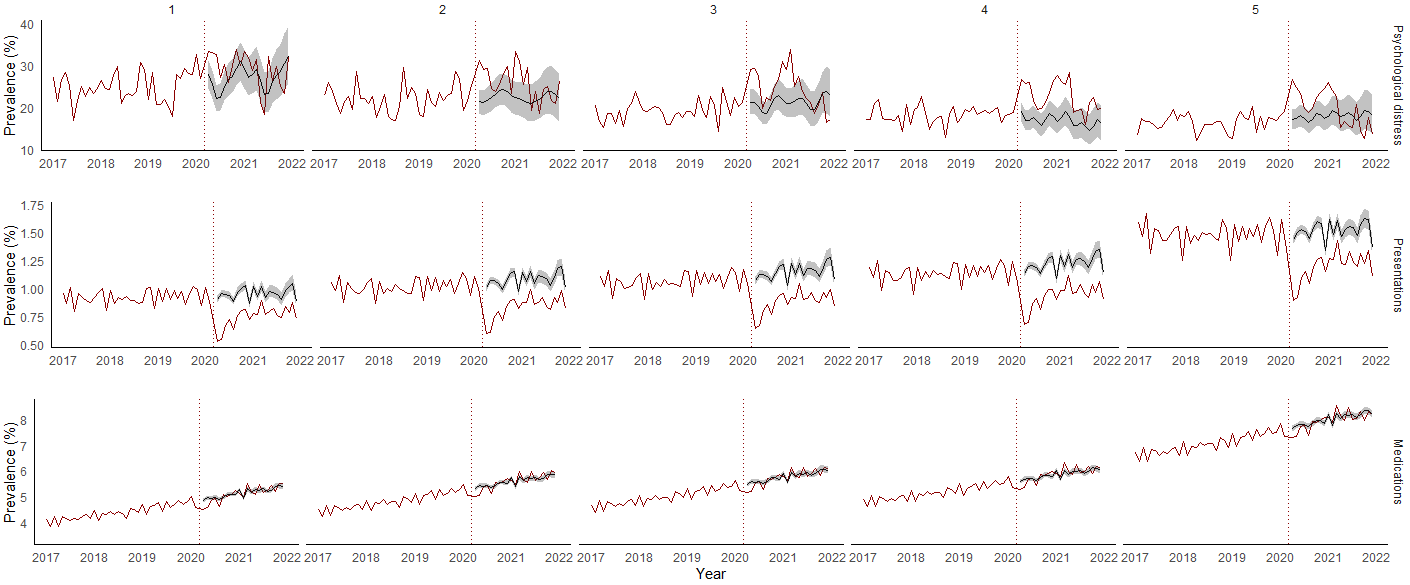


**Supplementary Figure S6: Prevalence Ratio (PR) between observed and expected values for IMD and region, by each outcome (psychological distress, primary care presentations for anxiety or depression, medications for anxiety or depression medications); by pandemic phase (1 (April - May 2020); P1 (June- September 2020); 2 (October 2020-February 2021); P2 (March – May 2021); 3 (June –December 2021))**

**
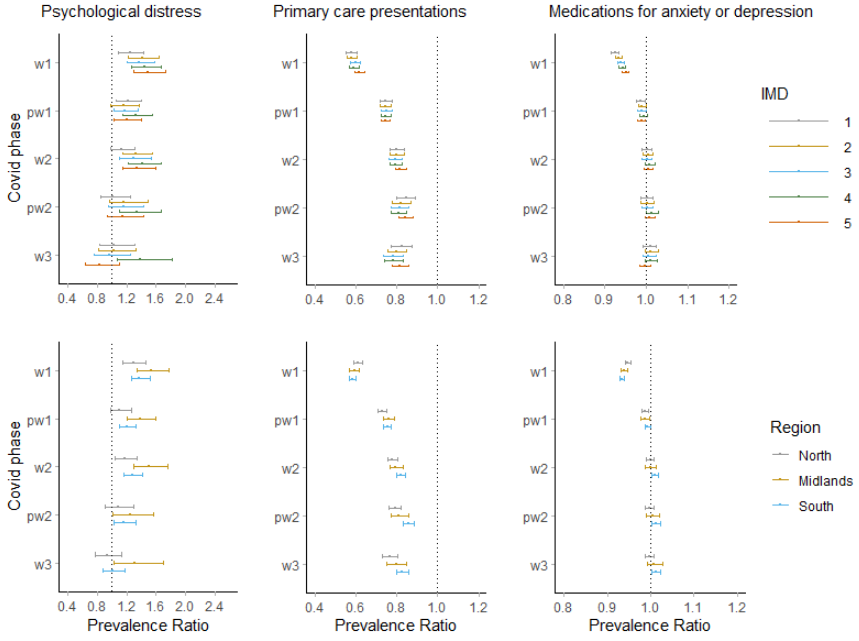
**

**Supplementary Table S9: results by region**

| **Subgroup** | **Cohort** | **Outcome** | **Period** | **Observed*** | **Expected*** | **Difference**  **[95% CI]** | **Ratio**  **[95% CI]** | **P-value** |
| --- | --- | --- | --- | --- | --- | --- | --- | --- |
| North | Survey | Psychological distress | 1^st^ Wave | 27.75 | 21.51  [19.29, 23.83] | 6.24  [3.28, 9.08] | 1.290  [1.141, 1.460] | <0.001 |
|  |  |  | Post 1^st^ Wave | 22.81 | 20.57  [18.36, 22.73] | 2.24  [-0.39, 4.97] | 1.109  [0.982, 1.261] | 0.071 |
|  |  |  | 2^nd^ Wave | 26.87 | 22.76  [20.15, 25.72] | 4.10  [1.04, 7.22] | 1.180  [1.041, 1.347] | 0.019 |
|  |  |  | Post 2^nd^ Wave | 24.09 | 22.19  [19.04, 25.71] | 1.89  [-2.13, 5.66] | 1.085  [0.915, 1.294] | 0.160 |
|  |  |  | 3^rd^ Wave | 20.79 | 22.18  [18.34, 26.53] | -1.38  [-6.06, 2.71] | 0.938  [0.769, 1.140] | 0.720 |
| Midlands |  |  | 1^st^ Wave | 30.81 | 20.08  [17.46, 22.57] | 10.73  [7.54, 13.95] | 1.534  [1.342, 1.771] | <0.001 |
|  |  |  | Post 1^st^ Wave | 26.03 | 18.72  [16.47, 21.23] | 7.31  [4.19, 10.23] | 1.390  [1.205, 1.599] | <0.001 |
|  |  |  | 2^nd^ Wave | 28.64 | 19.13  [16.43, 22.04] | 9.51  [6.20, 12.94] | 1.497  [1.289, 1.753] | <0.001 |
|  |  |  | Post 2^nd^ Wave | 25.83 | 20.70  [16.95, 24.67] | 5.13  [0.33, 9.77] | 1.248  [1.013, 1.558] | 0.043 |
|  |  |  | 3^rd^ Wave | 23.08 | 17.54  [14.07, 21.79] | 5.54  [0.74, 9.82] | 1.316  [1.032, 1.695] | 0.034 |
| South |  |  | 1^st^ Wave | 28.44 | 20.72  [19.10, 22.31] | 7.71  [5.94, 9.87] | 1.372  [1.272, 1.513] | <0.001 |
|  |  |  | Post 1^st^ Wave | 24.03 | 19.91  [18.26, 21.54] | 4.12  [2.18, 6.07] | 1.207  [1.103, 1.327] | <0.001 |
|  |  |  | 2^nd^ Wave | 28.36 | 22.18  [20.23, 24.19] | 6.17  [4.02, 8.42] | 1.278  [1.170, 1.410] | <0.001 |
|  |  |  | Post 2^nd^ Wave | 25.06 | 21.54  [19.16, 23.94] | 3.53  [0.65, 6.41] | 1.164  [1.026, 1.329] | 0.021 |
|  |  |  | 3^rd^ Wave | 21.22 | 20.96  [18.12, 23.82] | 0.26  [-2.78, 3.44] | 1.013  [0.882, 1.184] | 0.396 |
| North | Primary care | Presentations for anxiety or depression | 1^st^ Wave | 0.96 | 1.56  [1.53, 1.60] | -0.61  [-0.65, -0.57] | 0.611  [0.592, 0.631] | <0.001 |
|  |  |  | Post 1^st^ Wave | 1.16 | 1.59  [1.56, 1.64] | -0.43  [-0.47, -0.39] | 0.730  [0.709, 0.750] | <0.001 |
|  |  |  | 2^nd^ Wave | 1.28 | 1.64  [1.60, 1.69] | -0.36  [-0.42, -0.32] | 0.779  [0.754, 0.803] | <0.001 |
|  |  |  | Post 2^nd^ Wave | 1.32 | 1.67  [1.61, 1.73] | -0.35  [-0.42, -0.29] | 0.792  [0.759, 0.823] | <0.001 |
|  |  |  | 3^rd^ Wave | 1.29 | 1.68  [1.61, 1.76] | -0.39  [-0.47, -0.32] | 0.769  [0.730, 0.803] | <0.001 |
| Midlands |  |  | 1^st^ Wave | 0.70 | 1.18  [1.15, 1.22] | -0.48  [-0.52, -0.45] | 0.592  [0.568, 0.617] | <0.001 |
|  |  |  | Post 1^st^ Wave | 0.90 | 1.19  [1.15, 1.23] | -0.29  [-0.33, -0.25] | 0.760  [0.732, 0.788] | <0.001 |
|  |  |  | 2^nd^ Wave | 1.00 | 1.25  [1.20, 1.30] | -0.26  [-0.30, -0.21] | 0.796  [0.766, 0.830] | <0.001 |
|  |  |  | Post 2^nd^ Wave | 1.03 | 1.27  [1.21, 1.33] | -0.24  [-0.30, -0.18] | 0.812  [0.771, 0.857] | <0.001 |
|  |  |  | 3^rd^ Wave | 1.02 | 1.27  [1.20, 1.34] | -0.26  [-0.33, -0.18] | 0.798  [0.753, 0.848] | <0.001 |
| South |  |  | 1^st^ Wave | 0.55 | 0.94  [0.93, 0.96] | -0.39  [-0.41, -0.37] | 0.585  [0.569, 0.601] | <0.001 |
|  |  |  | Post 1^st^ Wave | 0.72 | 0.95  [0.94, 0.97] | -0.24  [-0.25, -0.22] | 0.753  [0.737, 0.770] | <0.001 |
|  |  |  | 2^nd^ Wave | 0.81 | 0.98  [0.96, 1.00] | -0.18  [-0.20, -0.15] | 0.819  [0.799, 0.841] | <0.001 |
|  |  |  | Post 2^nd^ Wave | 0.85 | 0.98  [0.96, 1.01] | -0.14  [-0.17, -0.11] | 0.859  [0.831, 0.887] | <0.001 |
|  |  |  | 3^rd^ Wave | 0.81 | 0.98  [0.95, 1.02] | -0.17  [-0.20, -0.14] | 0.826  [0.798, 0.857] | <0.001 |
| North |  | Medications for anxiety or depression | 1^st^ Wave | 7.42 | 7.83  [7.75, 7.94] | -0.41  [-0.47, -0.36] | 0.947  [0.941, 0.954] | <0.001 |
|  |  |  | Post 1^st^ Wave | 7.79 | 7.89  [7.81, 8.00] | -0.10  [-0.17, -0.05] | 0.987  [0.979, 0.994] | <0.001 |
|  |  |  | 2^nd^ Wave | 8.07 | 8.08  [8.00, 8.20] | -0.01  [-0.08, 0.05] | 0.999  [0.990, 1.007] | 0.352 |
|  |  |  | Post 2^nd^ Wave | 8.32 | 8.34  [8.23, 8.47] | -0.02  [-0.11, 0.06] | 0.998  [0.987, 1.008] | 0.308 |
|  |  |  | 3^rd^ Wave | 8.43 | 8.44  [8.33, 8.58] | -0.01  [-0.11, 0.07] | 0.998  [0.987, 1.008] | 0.327 |
| Midlands |  |  | 1^st^ Wave | 5.90 | 6.28  [6.19, 6.39] | -0.38  [-0.44, -0.33] | 0.939  [0.930, 0.947] | <0.001 |
|  |  |  | Post 1^st^ Wave | 6.28 | 6.36  [6.26, 6.46] | -0.08  [-0.14, -0.02] | 0.988  [0.977, 0.997] | 0.009 |
|  |  |  | 2^nd^ Wave | 6.53 | 6.54  [6.43, 6.64] | -0.01  [-0.08, 0.08] | 0.999  [0.988, 1.013] | 0.490 |
|  |  |  | Post 2^nd^ Wave | 6.75 | 6.72  [6.59, 6.84] | 0.03  [-0.07, 0.14] | 1.004  [0.990, 1.021] | 0.235 |
|  |  |  | 3^rd^ Wave | 6.87 | 6.81  [6.65, 6.93] | 0.05  [-0.05, 0.19] | 1.008  [0.993, 1.028] | 0.129 |
| South |  |  | 1^st^ Wave | 4.49 | 4.80  [4.76, 4.85] | -0.32  [-0.34, -0.29] | 0.934  [0.928, 0.940] | <0.001 |
|  |  |  | Post 1^st^ Wave | 4.81 | 4.85  [4.81, 4.90] | -0.03  [-0.07, -0.01] | 0.993  [0.986, 0.999] | 0.008 |
|  |  |  | 2^nd^ Wave | 5.02 | 4.97  [4.93, 5.02] | 0.05  [0.01, 0.08] | 1.010  [1.002, 1.017] | 0.005 |
|  |  |  | Post 2^nd^ Wave | 5.17 | 5.10  [5.05, 5.16] | 0.07  [0.02, 0.11] | 1.013  [1.003, 1.022] | 0.005 |
|  |  |  | 3^rd^ Wave | 5.21 | 5.15  [5.09, 5.20] | 0.07  [0.02, 0.11] | 1.013  [1.004, 1.022] | 0.003 |

# Supplementary Figure S7: Time-series plots by region


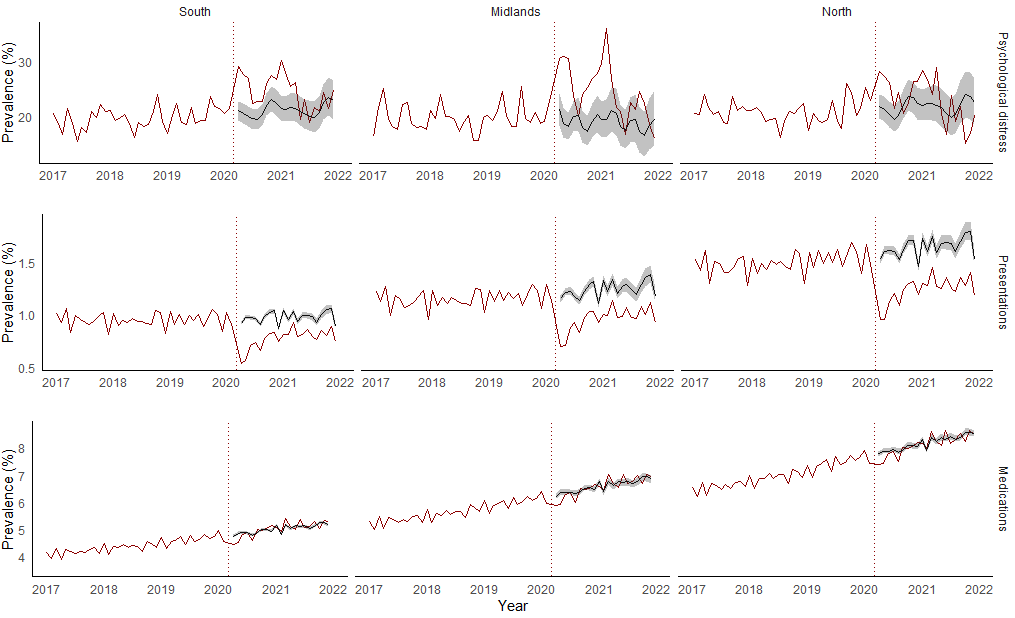


# Supplementary Table S10: Sensitivity analysis 1 changing definition of outcome in primary care cohort

| **Cohort** | **Outcome** | **Period** | **Observed** | **Expected** | **Difference**  **[95% CI]** | **Ratio**  **[95% CI]** | **P-value** |
| --- | --- | --- | --- | --- | --- | --- | --- |
| Primary care | Diagnosis only | 1^st^ Wave | 0.60 | 0.91  [0.89, 0.93] | -0.31  [-0.33, -0.29] | 0.659  [0.645, 0.676] | <0.001 |
|  |  | Post 1^st^ Wave | 0.75 | 0.91  [0.89, 0.93] | -0.16  [-0.18, -0.14] | 0.827  [0.808, 0.844] | <0.001 |
|  |  | 2^nd^ Wave | 0.84 | 0.94  [0.93, 0.96] | -0.11  [-0.13, -0.09] | 0.887  [0.867, 0.904] | <0.001 |
|  |  | Post 2^nd^ Wave | 0.88 | 0.94  [0.92, 0.96] | -0.06  [-0.08, -0.04] | 0.936  [0.914, 0.957] | <0.001 |
|  |  | 3^rd^ Wave | 0.84 | 0.94  [0.92, 0.96] | -0.10  [-0.12, -0.08] | 0.897  [0.876, 0.914] | <0.001 |
|  | Diagnosis & symptoms with history | 1^st^ Wave | 0.64 | 0.99  [0.98, 1.01] | -0.36  [-0.37, -0.35] | 0.639  [0.627, 0.651] | <0.001 |
|  |  | Post 1^st^ Wave | 0.80 | 1.00  [0.99, 1.01] | -0.20  [-0.21, -0.19] | 0.799  [0.787, 0.810] | <0.001 |
|  |  | 2^nd^ Wave | 0.89 | 1.04  [1.02, 1.05] | -0.15  [-0.16, -0.13] | 0.859  [0.847, 0.870] | <0.001 |
|  |  | Post 2^nd^ Wave | 0.93 | 1.03  [1.02, 1.05] | -0.10  [-0.12, -0.09] | 0.902  [0.887, 0.916] | <0.001 |
|  |  | 3^rd^ Wave | 0.90 | 1.03  [1.02, 1.05] | -0.14  [-0.15, -0.12] | 0.869  [0.856, 0.882] | <0.001 |

# Supplementary Figure S8: plots after changing definition of outcome in primary care cohort


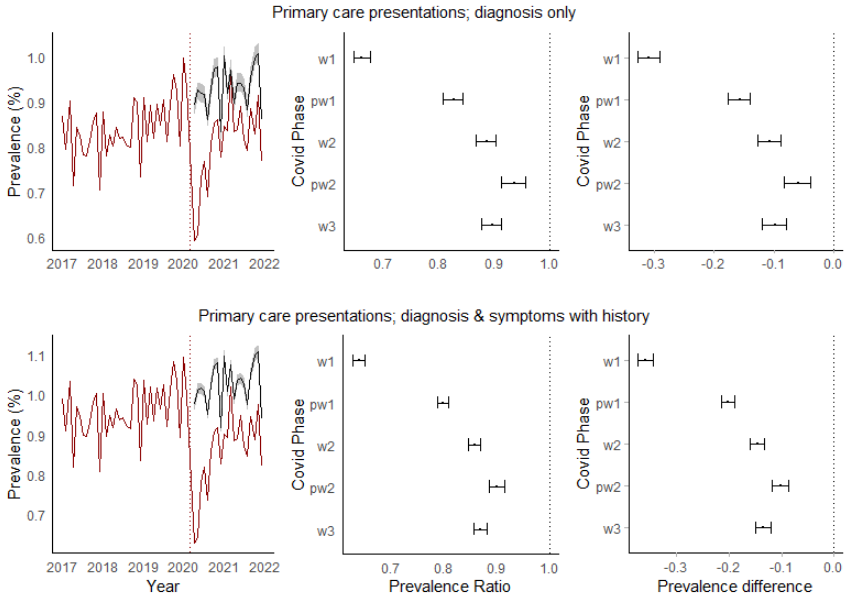


# Supplementary Figure S9: Sensitivity 2 Results from time-series analyses using the ARIMA model


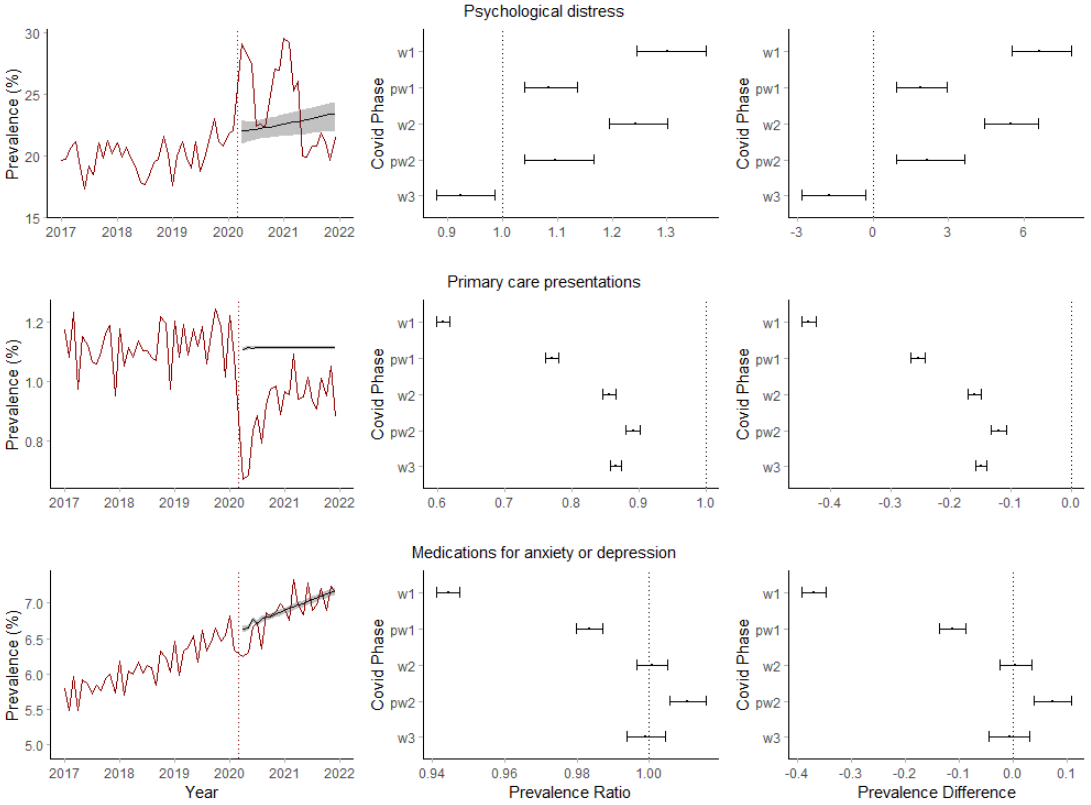


# Supplementary Table S11: post-hoc analysis, results for new and repeated prescriptions and presentations

| **Cohort** | **Outcome** | **Period** | **Observed** | **Expected** | **Difference**  **[95% CI]** | **Ratio**  **[95% CI]** | **P-value** |
| --- | --- | --- | --- | --- | --- | --- | --- |
| Primary care | New presentations | 1^st^ Wave | 0.29 | 0.53  [0.52, 0.53] | -0.24  [-0.25, -0.23] | 0.545  [0.531, 0.560] | <0.001 |
|  |  | Post 1^st^ Wave | 0.41 | 0.54  [0.53, 0.55] | -0.12  [-0.13, -0.11] | 0.772  [0.756, 0.788] | <0.001 |
|  |  | 2^nd^ Wave | 0.45 | 0.55  [0.53, 0.56] | -0.10  [-0.11, -0.09] | 0.812  [0.799, 0.837] | <0.001 |
|  |  | Post 2^nd^ Wave | 0.46 | 0.55  [0.54, 0.57] | -0.10  [-0.11, -0.08] | 0.828  [0.806, 0.859] | <0.001 |
|  |  | 3^rd^ Wave | 0.45 | 0.56  [0.53, 0.57] | -0.10  [-0.12, -0.08] | 0.814  [0.794, 0.854] | <0.001 |
|  | Repeated presentations | 1^st^ Wave | 9.35 | 13.69  [13.45, 13.92] | -4.34  [-4.65, -4.04] | 0.683  [0.665, 0.702] | <0.001 |
|  |  | Post 1^st^ Wave | 12.83 | 13.69  [13.38, 13.92] | -0.86  [-1.15, -0.52] | 0.937  [0.917, 0.961] | <0.001 |
|  |  | 2^nd^ Wave | 14.57 | 13.89  [13.48, 14.15] | 0.68  [0.39, 1.15] | 1.049  [1.028, 1.085] | <0.001 |
|  |  | Post 2^nd^ Wave | 14.26 | 13.90  [13.39, 14.28] | 0.36  [-0.09, 0.91] | 1.026  [0.994, 1.067] | 0.046 |
|  |  | 3^rd^ Wave | 13.57 | 13.77  [13.11, 14.18] | -0.20  [-0.65, 0.49] | 0.985  [0.954, 1.038] | 0.407 |
|  | New medications | 1^st^ Wave | 0.23 | 0.36  [0.36, 0.37] | -0.13  [-0.14, -0.13] | 0.634  [0.616, 0.653] | <0.001 |
|  |  | Post 1^st^ Wave | 0.31 | 0.37  [0.36, 0.37] | -0.06  [-0.06, -0.05] | 0.844  [0.828, 0.861] | <0.001 |
|  |  | 2^nd^ Wave | 0.33 | 0.38  [0.37, 0.38] | -0.04  [-0.05, -0.04] | 0.886  [0.870, 0.901] | <0.001 |
|  |  | Post 2^nd^ Wave | 0.33 | 0.38  [0.38, 0.39] | -0.05  [-0.06, -0.04] | 0.864  [0.845, 0.883] | <0.001 |
|  |  | 3^rd^ Wave | 0.34 | 0.38  [0.38, 0.39] | -0.05  [-0.05, -0.04] | 0.878  [0.864, 0.892] | <0.001 |
|  | Repeated medications | 1^st^ Wave | 56.30 | 58.56  [58.33, 58.81] | -2.26  [-2.49, -2.05] | 0.961  [0.958, 0.965] | <0.001 |
|  |  | Post 1^st^ Wave | 60.52 | 59.24  [58.94, 59.56] | 1.28  [0.96, 1.58] | 1.022  [1.016, 1.027] | <0.001 |
|  |  | 2^nd^ Wave | 61.78 | 60.15  [59.73, 60.55] | 1.63  [1.22, 2.04] | 1.027  [1.020, 1.034] | <0.001 |
|  |  | Post 2^nd^ Wave | 61.56 | 60.82  [60.37, 61.32] | 0.74  [0.24, 1.22] | 1.012  [1.004, 1.020] | 0.001 |
|  |  | 3^rd^ Wave | 61.42 | 60.85  [60.31, 61.44] | 0.57  [0.02, 1.12] | 1.009  [1.000, 1.019] | 0.032 |

# Supplementary Figure S10: time-series plots of new and repeated presentations and prescriptions


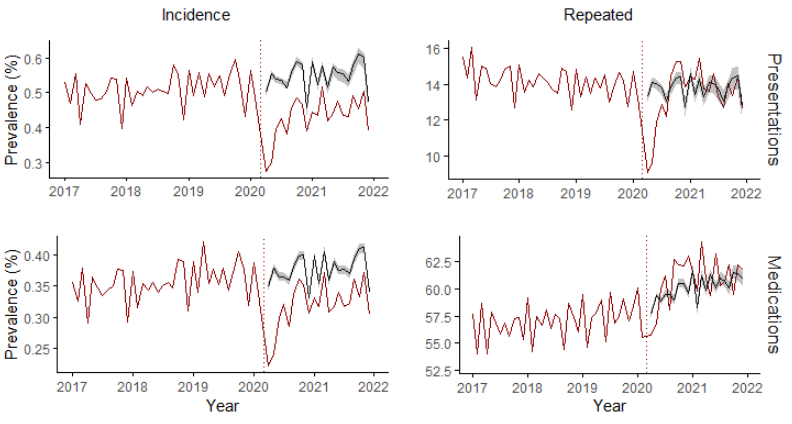


# References

1 Adir Y, Saliba W, Beurnier A, Humbert M. Asthma and COVID-19: an update. *Eur Respir Rev* 2021; **30**. DOI:10.1183/16000617.0152-2021.

2 Releases NC for ASNERCP. Air pollution falling UK cities, latest data shows. https://ncas.ac.uk/air-pollution-falling-across-uk-cities-latest-data-shows/ (accessed March 10, 2023).

3 Halpin DMG, Rabe AP, Loke WJ, *et al.* Epidemiology, Healthcare Resource Utilization, and Mortality of Asthma and COPD in COVID-19: A Systematic Literature Review and Meta-Analyses. *J Asthma Allergy* 2022; **15**: 811–25.

4 Kadambari S, Goldacre R, Morris E, Goldacre MJ, Pollard AJ. Indirect effects of the covid-19 pandemic on childhood infection in England: population based observational study. *BMJ* 2022; **376**. DOI:10.1136/bmj-2021-067519.

5 Joffres M, Falaschetti E, Gillespie C, *et al.* Hypertension prevalence, awareness, treatment and control in national surveys from England, the USA and Canada, and correlation with stroke and ischaemic heart disease mortality: a cross-sectional study Outcomes: Stroke and IHD mortality rates were plotted against countries’ specific prevalence data. 2007. DOI:10.1136/bmjopen-2013.

6 Lai H, Yang M, Sun M, *et al.* Risk of incident diabetes after COVID-19 infection: A systematic review and meta-analysis. Metabolism. 2022; **137**. DOI:10.1016/j.metabol.2022.155330.

7 Derksen VFAM, Kissel T, Lamers-Karnebeek FBG, *et al.* Onset of rheumatoid arthritis after COVID-19: Coincidence or connected? Ann. Rheum. Dis. 2021; **80**: 1096–8.

8 Butler CC, Hawking MKD, Quigley A, McNulty CAM. Incidence, severity, help seeking, and management of uncomplicated urinary tract infection: A population-based survey. Br. J. Gen. Pract. 2015; **65**: e702–7.

9 NHS Data Model and Dictionary. Person gender code. https://www.datadictionary.nhs.uk/attributes/person_gender_code.html#:~:text=National Code %27Not Known%27 means,as either male or female. (accessed March 10, 2023).

10 Mathur R, Palla L, Farmer RE, Chaturvedi N, Smeeth L. Ethnic differences in the severity and clinical management of type 2 diabetes at time of diagnosis: A cohort study in the UK Clinical Practice Research Datalink. *Diabetes Res Clin Pract* 2020; **160**. DOI:10.1016/j.diabres.2020.108006.

11 Wolf A, Dedman D, Campbell J, *et al.* Data resource profile: Clinical Practice Research Datalink (CPRD) Aurum. *Int J Epidemiol* 2019; **48**: 1740-1740G.

12 Bhaskaran K, Gasparrini A, Hajat S, Smeeth L, Armstrong B. Time series regression studies in environmental epidemiology. *Int J Epidemiol* 2013; **42**: 1187–95.
